# Application of a novel deep eutectic solvent as a capable and new catalyst for the synthesis of tetrahydropyridines and 1,3-thiazolidin-4-ones

**DOI:** 10.1038/s41598-023-32882-0

**Published:** 2023-04-10

**Authors:** Hadis Goudarzi, Davood Habibi, Arezo Monem

**Affiliations:** Department of Organic Chemistry, Faculty of Chemistry, Bu-Ali Sina University, Hamedan, Iran

**Keywords:** Catalysis, Green chemistry, Organic chemistry, Chemistry

## Abstract

A novel deep eutectic solvent (ETPP-Br/THF-TCA-DES) was prepared by a mixture of ethyl triphenylphosphonium bromide (ETPP-Br) and tetrahydrofuran-2,3,4,5-tetra-carboxylic acid (THF-TCA, mole ratio 7:3), characterized by FT-IR, TGA/DTA, densitometer, eutectic point, and ^1^H NMR techniques and used as a capable and new catalyst for the synthesis of two sets of compounds: (1) the four new **[a(1–4)]** and the eleven **[a(5–15)]** known alkyl 1,2,6-trisubstituted-4-[(hetero)arylamino]-1,2,5,6-tetrahydropyridine-3-carboxylates and (2) the two new **[b(1–2)]** and the eight **[b(3–10)]** known 1,3-thiazolidin-4-ones in DES with short reaction time, high yields, and easy recycling and separation of the DES catalyst. There is a nice consistency between the proposed structure of the DES compound, the integration values of the ^1^H NMR peaks and the ratio of ETPP-Br to THF-TCA obtained from the eutectic point phase diagram. Also, the decrease in splitting patterns of the peaks in DES, compared to the two starting materials can be the good evidence of the hydrogen bond formation between the two components.

## Introduction

In the context of green chemistry, solvents occupy a strategic position. The green solvents must meet different properties such as eco-friendly, non-toxicity, availability, recyclability, low flammability and affordable^1^. Deep eutectic solvents (DESs) are solvents that, in addition to having the properties of green solvents such as low melting point, stability towards moisture and air, low vapor pressure and high thermal stability, can be used as an alternative to ionic liquids^2,3^. The concept of DES was first described by Abbot and coworkers in 2003. Generally, a DES is a type of solvent composed of a mixing of two or more components under simple operation that forms a compound with a much lower melting point than either of the individual components^4,5^. DESs are usually eutectic mixtures of ammonium or phosphonium-based salts as hydrogen-bond acceptors (HBA) and alcohols, amines, acids, or other compounds as hydrogen-bond donors (HBD) and have a melting point much lower than that of the individual components^6–9^.

Multicomponent reactions are described by three or more reactants that are combined in a one-pot process to produce a single product^10^. They are economically and environmentally very important because multi-step synthesis usually produces a lot of waste and it is difficult to separate them after each step^11,12^.

Heterocycles are found in numerous natural products such as alkaloids, drug candidates and drugs^13–15^, and identified as an important class of therapeutic agents in the treatment of cancer metastasis^16,17^, schizophrenia^18,19^, Parkinson’s disease^20,21^, obesity, and diabetes^22,23^. Also, nitrogen containing heterocycles are abundant in synthetic bioactive molecules as well as in various natural products^24^ which exhibit a plethora of biological properties such as analgesic^25^, anticonvulsant^26^, antimalarial^27^, hyperglycemic^28^, nicotinic^29^ and anti-influenza^30^.

In addition, five-membered heterocycles with two heteroatoms have attracted special attention, which have proven applications in medicinal chemistry^31^. Specifically, 1,3-thia-zolidin-4-ones are important class of heterocyclic compounds due to their biological profiles like anti-tubercular^32^, anti-microbial^33^, anti-inflammatory^34^, anti-histaminic^35^, anti-hepatitis^36^, HIV inhibitors^37^, anti-hyper-glycemic^38^, anti-hypertensive^39^, anti-convulsant^40^, anti-fungal^41^, anti-oxidant^42^, and anti-proliferative^43^ activities.

**Figure 1:**
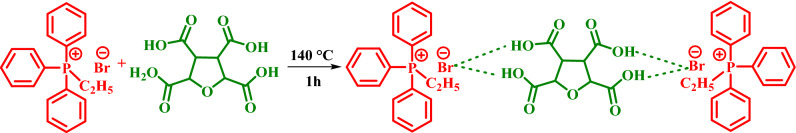
The proposed structure for ETPP-Br/THF-TCA-DES.

Herein, we would like to report the preparation of a novel ethyl triphenylphosphonium bromide-based Deep Eutectic Solvent (ETPP-Br/THF-TCA-DES) from the reaction of ethyl triphenylphosphonium-bromide and tetrahydrofuran-2,3,4,5-tetracarboxylic acid (Fig. 1).

**Figure 2:**
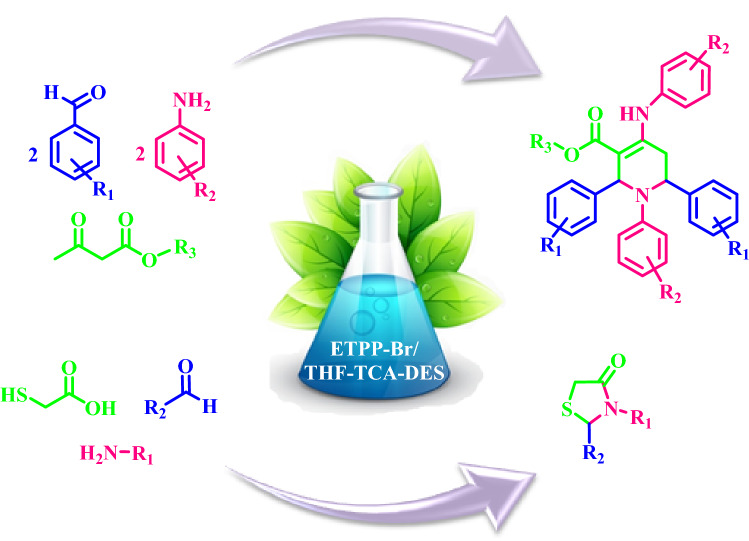
Synthesis of **a(1–15)** and **b(1–10)**.

Then, ETPP-Br/THF-TCA-DES was used as a capable and novel catalyst for the synthesis of the two series of the following compounds:

1. alkyl 1,2,6-trisubstituted-4-[(hetero)arylamino]-1,2,5,6-tetrahydropyridine-3-carboxylates **a(1–15)**, and
2. 1,3-thiazolidin-4-ones **b(1–10)** with high yields and appropriate times in DES (Fig. 2).

## Experimental

### Materials

Explanation about all reagents, solvents, chemicals, and the scientific devices are available in Supporting Information.

### General procedure for preparation of the ETPP-Br/THF-TCA-DES catalyst

The mixture (molar ratio 7:3) of ethyl triphenylphosphonium-bromide (ETPP-Br) and tetra-hydrofuran-2,3,4,5-tetracarboxylic acid (THF-TCA) was heated (140 °C) and stirred until a homogeneous liquid obtained and used as a catalyst for the preparation of **a(1–15)** and **b(1–10)**.

### General procedure for the synthesis of a(1–15)

A mixture of *β*-ketoester (1 mmol), an aniline derivative (2 mmol) and ETPP-Br/THF-TCA-DES (0.5 mmol) was stirred and heated at 80 °C, followed by the addition of aromatic aldehyde (2 mmol). After completion of the reaction (TLC), the mixture was diluted with water (10 mL) and chloroform (10 mL) and shaked. The aqueous layer which contains DES was separated from the organic layer by simple liquid–liquid extraction and the organic layer dried and recrystallized in ethanol to afford the pure product.

### General procedure for the synthesis of b(1–10)

A mixture of aldehyde (1 mmol), aromatic amine (1 mmol), thioglycolic acid (0.138 g, 1.5 mmol) and ETPP-Br/THF-TCA-DES (0.16 mmol) was stirred and heated at 90 °C. After completion of the reaction (TLC), the mixture was diluted with water (5 mL) and ethyl acetate (10 mL) and shaked. The aqueous layer which contains DES was separated from the organic layer by simple liquid–liquid extraction and the organic layer dried and recrystallized in the *n*-hexane/ethyl acetate mixture or purified by plate (*n*-hexane:ethyl acetate ratio: 8:2).

## Results and discussion

### Synthesis of the ETPP-Br/THF-TCA-DES

The mixture of ETPP-Br and THF-TCA (mole ratio 7:3) was heated at 140 °C and stirred until a homogeneous liquid was obtained and used as a catalyst.

### Characterization of ETPP-Br/THF-TCA-DES

The prepared DES compound was characterized by Fourier transform infra-red (FT-IR), nuclear magnetic resonance spectroscopy (NMR), thermo gravimetric analysis-differential thermal analysis (TGA-DTA), Densitometer and eutectic points.

#### Characterization by FT-IR

**Figure 3:**
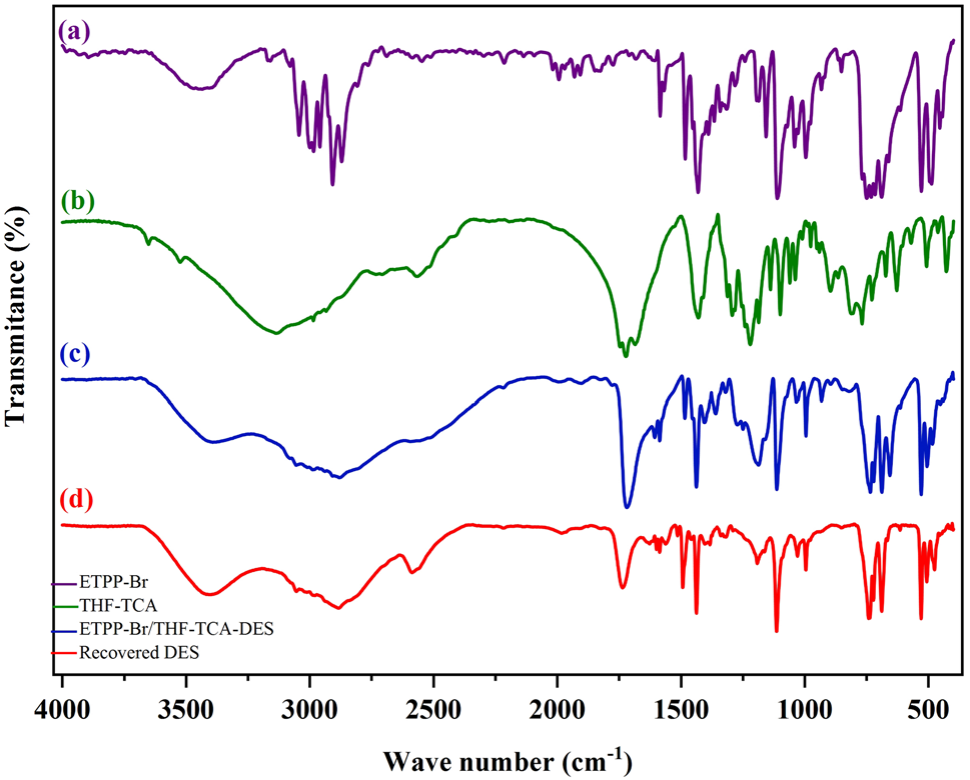
The FT-IR spectra of (**a**), (**b**), (**c**), and (**d**).

Figure 3 shows the IR spectra of ETPP-Br (a), THF-TCA (b), ETPP-Br/THF-TCA-DES (c), and the recovered DES catalyst (d).

In spectrum (a), the peaks at about 2900–3100 cm^−1^ are related to the aromatic and aliphatic hydrogens, and the peaks at about 750 and 1480 cm^−1^ are related to the C–P bonds, respectively. In spectrum (b), the peaks at 3600 and 1735 cm^−1^ are related to the O–H and C=O of the –COOH group, respectively. In the (c) spectrum, the indicated peaks can be seen in both (a) and (b) spectra, which confirm the structure of the DES catalyst. To confirm the structure of the recovered DES, the corresponding IR spectrum was recorded which shows that there is no significant difference between the original (fresh) and the recovered (used) IR spectra.

#### Characterization by eutectic points

**Figure 4:**
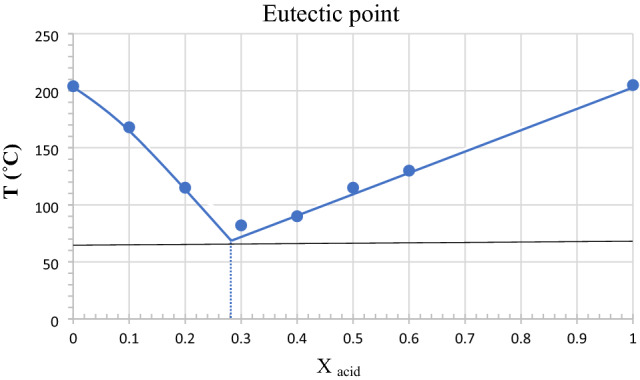
The eutectic points phase diagram of ETPP-Br/THF-TCA-DES.

To check the best ratio and the chemical composition of the ethyl triphenylphosphonium bromide to tetrahydrofuran-2,3,4,5-tetracarboxylic acid mixture, the eutectic point experiment was performed. In this experiment, ten different portions (100–0%) of ethyl triphenyl-phosphonium bromide with a melting point of 203 °C were added to the ten different portions (0–100%) of tetrahydrofuran-2,3,4,5-tetracarboxylic acid and the obtained melting points were recorded each time. Figure 4 shows the eutectic points phase diagram of ETPP-Br/THF-TCA-DES indicating that the best-obtained ratio of ETPP-Br to THF-TCA is about 7 to 3 (almost 2:1).

#### Characterization by ^1^H NMR

**Figure 5:**
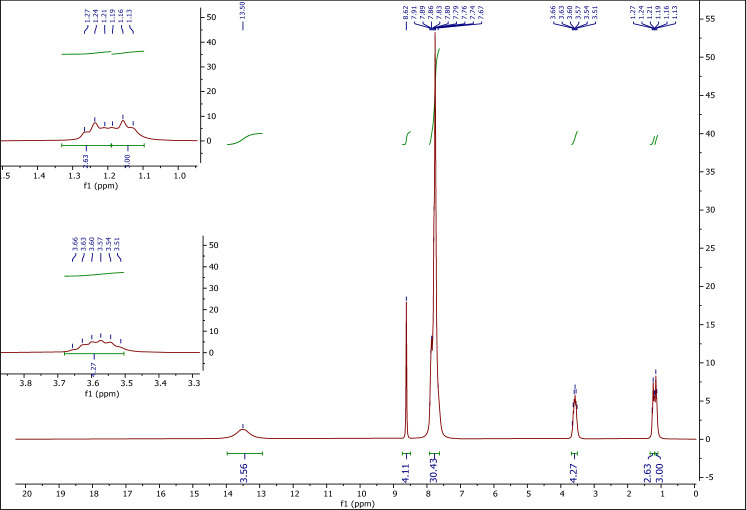
The ^1^H NMR of ETPP-Br/THF-TCA-DES.

Figure 5 shows the ^1^H NMR spectrum of ETPP-Br/THF-TCA-DES. The peak at 1.13–1.27 (t, 6H) ppm is related to the CH_3_ hydrogens, the peak at 3.51–3.66 (4H) ppm is related to the CH_2_ hydrogens, the peak at 7.67–7.91 (m, 30H) ppm is related to the phenyl ring hydrogens, the peak at 8.62 (s, 4H) ppm is related to the THF cycle hydrogens, and the peak at 13.50 (s, 4H) ppm is related to the –COOH acidic hydrogens. The decrease in the splitting patterns of the peaks in DES, compared to the starting materials can be a very nice evidence of hydrogen bonds formation between the two components which caused a gap between the phenyl groups in tetrahydrofuran-2,3,4,5-tetracarboxylic acid and the ethyl groups in ethyl triphenyl-phosphonium-bromide, so the long-range coupling is no longer possible.

Also, there is a nice consistency between the proposed structure for the ETPP-Br/THF-TCA-DES compound (Fig. 1) and the corresponding integration values of the ^1^H NMR peaks (Fig. 5). The ratio of the peak integration values of the Hs of the CH_3_ groups, the Hs of the CH_2_ groups, the Hs of the phenyl groups, the acidic Hs of the THF tetra-acid, and the Hs of the THF cycle is 6 (5.63) to 4 to 30 to 4 (3.56) to 4, indicating the presence of the six hydrogens of the two CH_3_ groups, the four hydrogens of the two CH_2_ groups, the thirty hydrogens of the six phenyl groups, the four acidic hydrogens and the four hydrogens of the THF cycle.

Incidentally, the results obtained from the FT-IR spectrum (Fig. 3), the eutectic points phase diagram (Fig. 4) and the ^1^H NMR integration values (Fig. 5) are almost in the same direction (the ratio of ETPP-Br to THF-TCA is almost two to one), confirming the proposed structure for the ETPP-Br/THF-TCA-DES compound (Fig. 1).

#### Characterization by TGA-DTA

**Figure 6:**
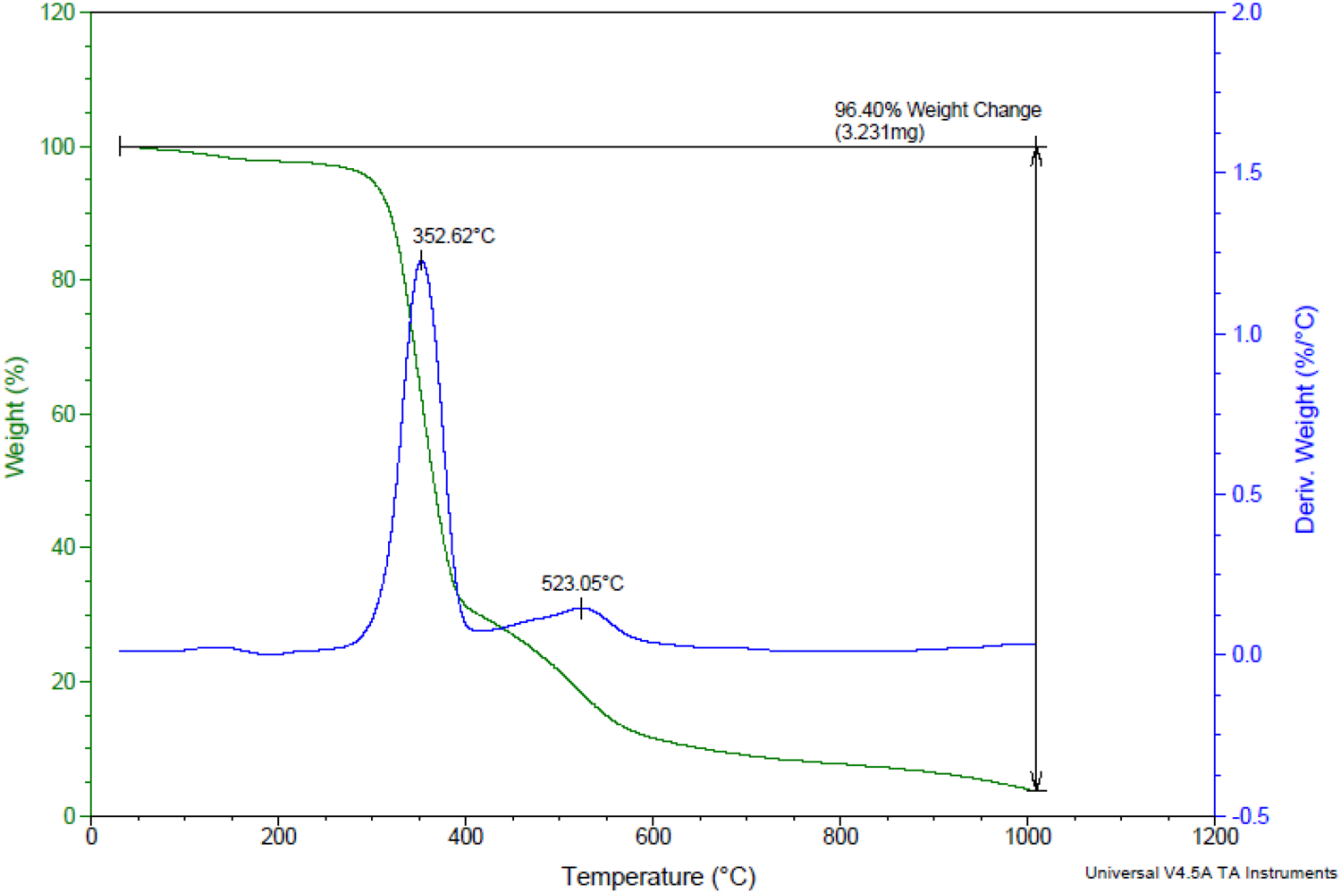
The TGA-DTA patterns of ETPP-Br/THF-TCA-DES.

To investigate the stability and thermal behavior of ETPP-Br/THF-TCA-DES, the TGA-DTA analysis was performed (Fig. 6). The curve shows the four weight losses which are probably related to the removal of water, organic solvents, acidic compound, and breakdown of the hydrogen bond and the ionic bonds, respectively. These results indicate the high thermal stability of ETPP-Br/THF-TCA-DES.

#### Characterization by densitometer

Most of DESs are denser than water, for example, the density of the ZnCl_2_-HBD-DES is about 1.3 g/cm^3^. So, a sample with a certain weight is mixed with a certain volume of water, then by using the formula (P = P_w_/1 − m_w_/m_d_), the density is calculated. The density of ETPP-Br/THF-TCA-DES is about 1.3215 g/mL (P = P_w_/1 − m_w_/m_d_ = 0.99618/1–0.0355/0.1442 = 1.3215 g/mL) (Table 1).

**Table 1:** The ETPP-Br/THF-TCA-DES catalyst density data table.

Mass of 1.0 mL of water (g, P_w_)	Wet sample mass (g, M_w_)	Sample dry mass (g, M_d_)
0.99618	0.0355	0.1442

### Optimization of the reaction conditions in the synthesis of a(1–15)

The effect of different solvents, temperatures, and amounts of the DES catalyst was investigated on model reaction (reaction of ethyl acetoacetate, aniline, and 4-chlorobenz-aldehydes) to optimize the reaction conditions.

First, the model reaction was performed in ethanol, water, water/ethanol, ethyl acetate, *n*-hexane, and the DES conditions which the DES conditions showed to be the best (Table 2).

**Table 2:** Optimization of solvent by DES.

Entry	Temp. (°C)	Solvent	Yield (%)
1	Reflux	Ethanol	40
2	Reflux	Water	–
3	Reflux	EtOH + H_2_O	–
4	Reflux	Ethyl acetate	32
5	Reflux	*n*-Hexane	–
**6**	**80**	**DES**	**90**

Significant values are in [bold].

Then, the model reaction was conducted in different temperatures which the temperature of 80 °C had the best efficiency (Table 3).

**Table 3:** Optimization of temperature by DES in DES.

Entry	Temp. (°C)	Yield (%)
**1**	**80**	**90**
2	90	78
3	100	62
4	110	65

Significant values are in [bold].

Finally, the model reaction was carried out with different amounts of the DES catalyst which 0.5 mmol of the catalyst had the best yield (Table 4).

**Table 4:** Optimization of the DES amount at 80 °C.

Entry	DES (mmol)^a^	Yield (%)
1	0.25	60
**2**	**0.5**	**78**
3	0.75	65
4	1.00	62
5	0.00	No reaction

Calculation: MW of Ph_3_PEtBr = 371 and MW of tetra-acid = 248, so the MW of DES = 2 × 371 + 248 = 990. As a result, 0.5 g of DES is equal to 0.5 ÷ 990 × 1000 = about 0.5 mol.

Significant values are in [bold].

^a^Exceptionally, in the case of this catalyst (DES), the amount of mg of the used catalyst is almost the same as its mmole.

Overall, we concluded that the best condition was found to be the 2:2:1 mol ratio of aniline, 4-chlorobenzaldehyde and ethyl acetoacetate with 0.5 mmol of the DES catalyst at 80 °C in appropriate times.

### Synthesis of diverse a(1–15)

Based on the results obtained from the optimized conditions (synthesis of **a6**), **a(1–15)** were synthesized from the reaction of *β*-ketoester, aniline, and aldehyde at 80 °C in DES with high yields and appropriate reaction times (Table 5).

**Table 5:** Synthesis of **a(1–15)** by the DES catalyst.


Entry	Aldehyde	Amine	R_3_	Product	Time (h)	Yield (%)	M.P. °C (Found, lit.) ref	TON^a^	TOF^a^
1	4-iso-Pr-C_7_H_5_O	4-Cl-C_6_H_7_N	Et	**a1**	2	65	230–232 NEW	130	65
2	4-iso-Pr-C_7_H_5_O	4-Br-C_6_H_7_N	Et	**a2**	1	63	237–240 NEW	126	126
3	3-NO_2_-C_7_H_5_O	4-Me-C_6_H_8_N_2_	Et	**a3**	4	72	174–176 NEW	144	36
4	3-NO_2_-C_7_H_5_O	4-Cl-C_6_H_7_N	Me	**a4**	2	60	201–203 NEW	120	60
5	C_7_H_6_O	C_6_H_7_N	Et	**a5**	1.5	73	174–176 174–176^44^	146	97.33
6	4-Cl-C_7_H_5_O	C_6_H_7_N	Et	**a6**	3	90	237–239 230–232^45^	184	61.33
7	C_7_H_6_O	4-Cl-C_6_H_7_N	Et	**a7**	2	95	221–224 229–230^46^	190	95
8	4-Cl-C_7_H_5_O	4-Cl-C_6_H_7_N	Et	**a8**	2.5	76	215–218 214–215^47^	152	60.8
9	3-NO_2_-C_7_H_5_O	4-Cl-C_6_H_7_N	Et	**a9**	2	94	175–178 161–162^48^	188	94
10	3-Br-C_7_H_5_O	4-Cl-C_6_H_7_N	Et	**a10**	2	92	211–213 239–24^48^	184	92
11	C_7_H_6_O	4-Br-C_6_H_7_N	Et	**a11**	1.5	85	223–227 228–230^49^	170	113.3
12	4-Cl-C_7_H_5_O	4-Br-C_6_H_7_N	Et	**a12**	2.5	74	202–205 201–203^50^	148	59.2
13	3-NO_2_-C_7_H_5_O	4-Br-C_6_H_7_N	Et	**a13**	2	93	180–183 194–195^48^	186	93
14	3-Br-C_7_H_5_O	4-Br-C_6_H_7_N	Et	**a14**	3.5	71	218–220 165–166^48^	142	40.5
15	C_7_H_6_O	4-I-C_6_H_7_N	Et	**a15**	2.5	80	234–237 243–245^51^	160	64

^a^TON = yield/amount of catalyst (mole), TOF = TON/time.

### Spectral data^44–51^

#### Ethyl 1-(4-chlorophenyl)-4-((4-chlorophenyl)amino)-2,6-bis(4-isopropylphenyl)-1,2,5,6-tetrahydropyridine-3-carboxylate (a1)

White solid, M.P.: 230–232 °C; IR (KBr) ν: 3242, 2961, 2868, 1645, 1604, 1493, 1368, 1318, 1256, 1176, 1070 and 754 cm^−1^; ^1^H NMR (250 MHz, CDCl_3_) δ = 10.22 (s, 1H), 7.24–6.85 (m, 12H), 6.58–6.25 (m, 3H), 6.06 (d, *J* = 8.2 Hz, 2H), 5.05 (s, 1H), 4.39 (d, *J* = 37.4 Hz, 2H), 2.89 (dd, *J* = 14.9, 7.8 Hz, 2H), 2.69 (dd, *J* = 41.3, 10.6 Hz, 2H), 1.46 (t, *J* = 7.1 Hz, 3H), 1.23 (d, *J* = 6.4 Hz, 12H); ^13^C NMR (62.5 MHz, CDCl_3_) δ = 168.21, 155.59, 148.22, 146.91, 145.57, 140.36, 139.81, 136.45, 128.83, 128.63, 127.22, 126.79, 126.34, 126.14, 120.86, 116.53, 113.90, 98.69, 77.49, 76.98, 76.48, 59.79, 58.19, 54.87, 33.75, 33.56, 33.43, 23.96 and 14.72; MW = 626; MS: m/z = 626 [M^+^], 625 (base peak).

#### Ethyl 1-(4-bromophenyl)-4-((4-bromophenyl)amino)-2,6-bis(4-isopropylphenyl)-1,2,5,6-tetrahydropyridine-3-carboxylate (a2)

White solid, M.P.: 237–240 °C; IR (KBr) ν: 3241, 2961, 2869, 1645, 1602, 1489, 1318, 1255, 1067, 1010, 827 and 799 cm^−1^; ^1^H NMR (250 MHz, CDCl_3_) δ = 10.22 (s, 1H), 7.13 (dq, *J* = 14.8, 7.7 Hz, 12H), 6.58–6.21 (m, 3H), 6.02 (d, *J* = 8.1 Hz, 2H), 5.06 (d, *J* = 5.4 Hz, 1H), 4.63–4.07 (m, 2H), 3.00–2.83 (m, 2H), 2.73 (d, *J* = 35.8 Hz, 2H), 1.47 (t, *J* = 7.1 Hz, 3H), 1.23 (d, *J* = 6.3 Hz, 12H); ^13^C NMR (62.5 MHz, CDCl_3_) δ = 168.22, 155.45, 148.24, 146.96, 145.99, 140.28, 139.69, 136.96, 131.83, 131.50, 127.50, 126.81, 126.36, 126.12, 119.99, 119.10, 116.97, 114.49, 108.13, 98.80, 59.82, 58.16, 54.85, 33.77, 33.58, 33.39, 23.97 and 14.74; MW = 716; MS: m/z = 716 [M^+^], 596.9 (base peak).

#### Ethyl 2,6-bis(3-nitrophenyl)-1-(*p*-tolyl)-4-(*p*-tolylamino)-1,2,5,6-tetrahydropyridine-3-carboxylate (a3)

Yellow solid, M.P.: 174–176 °C; IR (KBr) ν: 3215, 2974, 2915, 1653, 1593, 1520, 1348, 1317, 1255, 1070, 785 and 702 cm^−1^; ^1^H NMR (250 MHz, CDCl_3_) δ = 10.27 (s, 1H), 8.30 (s, 1H), 8.09 (d, *J* = 8.2 Hz, 2H), 7.94 (d, *J* = 5.7 Hz, 1H), 7.63 (d, *J* = 7.8 Hz, 1H), 7.45 (q, *J* = 7.0, 5.2 Hz, 3H), 6.90 (q, *J* = 7.5, 6.4 Hz, 4H), 6.43 (d, *J* = 5.6 Hz, 1H), 6.32 (dd, *J* = 16.7, 7.8 Hz, 4H), 5.27 (d, *J* = 4.5 Hz, 1H), 4.62–4.23 (m, 2H), 2.85 (d, *J* = 4.3 Hz, 2H), 2.27 (s, 3H), 2.17 (s, 3H), 1.51 (t, *J* = 7.2 Hz, 3H); ^13^C NMR (62.5 MHz, CDCl_3_) δ = 167.69, 155.61, 148.62, 146.70, 144.72, 143.57, 136.45, 134.52, 132.64, 132.35, 129.82, 129.66, 129.18, 126.77, 125.67, 122.37, 121.71, 121.43, 113.17, 96.45, 60.13, 57.02, 55.32, 33.66, 20.86, 20.09 and 14.77; MW = 592; MS: m/z = 592 [M^+^], 591 (base peak).

#### Methyl 1-(4-chlorophenyl)-4-((4-chlorophenyl)amino)-2,6-bis(3-nitrophenyl)-1,2,5,6-tetra- hydropyridine-3-carboxylate (a4)

White solid, M.P.: 201–203 °C; IR (KBr) ν: 3224, 3073, 2949, 1660, 1605, 1524, 1493, 1349, 1320, 1258, 1190, 803 and 733 cm^−1^; ^1^H NMR (250 MHz, CDCl_3_) δ = 10.28 (s, 1H), 8.46–7.82 (m, 4H), 7.82–7.33 (m, 4H), 7.33–6.89 (m, 4H), 6.61–6.14 (m, 5H), 5.30 (s, 1H), 3.99 (s, 3H), 2.86 (m, *J* = 5.7, 3.0 Hz, 2H); ^13^C NMR (62.5 MHz, CDCl_3_) δ = 167.88, 154.74, 148.61, 145.56, 144.23, 143.82, 135.64, 132.37, 129.85, 129.36, 129.21, 126.66, 122.90, 122.73, 122.08, 121.31, 114.24, 97.29, 57.01, 55.32, 51.60 and 33.65; MW = 618; MS: m/z = 618 [M^+^], 581 (base peak).

#### Ethyl 1,2,6-triphenyl-4-(phenylamino)-1,2,5,6-tetrahydropyridine-3-carboxylate (a5)

White solid, M.P.: 174–176 °C; IR (KBr) ν: 3248, 3457, 2981, 2872, 1652, 1593, 1500, 1373, 1252, 1172, 1070, 750 and 699 cm^−1^; ^1^H NMR (250 MHz, CDCl_3_) δ = 10.29 (s, 1H), 7.32 (s, 6H), 7.22–6.96 (m, 8H), 6.71–6.39 (m, 5H), 6.37–6.11 (m, 2H), 5.14 (d, *J* = 5.1 Hz, 1H), 4.63–4.19 (m, 2H), 3.00–2.64 (m, 2H), 1.47 (t, *J* = 7.1 Hz, 3H).

#### Ethyl 2,6-bis(4-chlorophenyl)-1-phenyl-4-(phenylamino)-1,2,5,6-tetrahydropyridine-3-carboxylate (a6)

Cream solid, M.P.: 237–239 °C; IR (KBr) ν: 3238, 3060, 2978, 2872, 1650, 1595, 1500, 1368, 1251, 1175, 1067, 748 and 691 cm^−1^; ^1^H NMR (250 MHz, CDCl_3_) δ = 10.30 (s, 1H), 7.39–7.01 (m, 13H), 6.65 (t, *J* = 7.2 Hz, 1H), 6.57–6.31 (m, 5H), 5.09 (d, *J* = 4.3 Hz, 1H), 4.59–4.16 (m, 2H), 2.79 (dd, *J* = 8.3, 4.2 Hz, 2H), 1.45 (t, *J* = 5.9, 4.6 Hz, 3H).

#### Ethyl 1-(4-chlorophenyl)-4-((4-chlorophenyl)amino)-2,6-diphenyl-1,2,5,6-tetrahydro-pyridine-3-carboxylate (a7)

White solid, M.P.: 221–224 °C; IR (KBr) ν: 3241, 3093, 2978, 2856, 1646, 1603, 1493, 1372, 1255, 1178, 1071, 727 and 698 cm^−1^; ^1^H NMR (250 MHz, CDCl_3_) δ = 10.24 (s, 1H), 7.38 (s, 4H), 7.27–6.93 (m, 9H), 6.59–6.28 (m, 4H), 6.17 (d, *J* = 8.2 Hz, 2H), 5.12 (s, 1H), 4.40 (ddt, *J* = 28.2, 10.8, 6.2 Hz, 2H), 2.99–2.57 (m, 2H), 1.47 (t, *J* = 7.1 Hz, 3H).

#### Ethyl 1,2,6-tris(4-chlorophenyl)-4-((4-chlorophenyl)amino)-1,2,5,6-tetrahydropyridine-3-carboxylate (a8)

White solid, M.P.: 215–218 °C; IR (KBr) ν: 3238, 3068, 2982, 2968, 1653, 1601, 1495, 1369, 1330, 1256, 1090 and 810 cm^−1^; ^1^H NMR (250 MHz, CDCl_3_) δ = 10.25 (s, 1H), 7.33 (s, 1H), 7.26–6.94 (m, 11H), 6.33 (dd, *J* = 16.6, 7.6 Hz, 5H), 5.06 (d, *J* = 5.0 Hz, 1H), 4.39 (dq, *J* = 25.4, 9.2 Hz, 2H), 2.91–2.56 (m, 2H), 1.45 (t, *J* = 7.2 Hz, 3H).

#### Ethyl 1-(4-chlorophenyl)-4-((4-chlorophenyl)amino)-2,6-bis(3-nitrophenyl)-1,2,5,6-tetra-hydropyridine-3-carboxylate (a9)

Yellow solid, M.P.: 175–178 °C; IR (KBr) ν: 3241, 3178, 2976, 2865, 1655, 1592, 1522, 1496, 1350, 1247, 1176, 1067, 810 and 732 cm^−1^; ^1^H NMR (250 MHz, CDCl_3_) δ = 10.34 (s, 1H), 8.38–7.88 (m, 4H), 7.70–7.35 (m, 4H), 7.20–6.91 (m, 4H), 6.41 (s, 1H), 6.40–6.21 (m, 4H), 5.30 (s, 1H), 4.68–4.24 (m, 2H), 2.86 (t, *J* = 4.9 Hz, 2H), 1.52 (t, *J* = 7.1 Hz, 3H).

#### Ethyl 2,6-bis(3-bromophenyl)-1-(4-chlorophenyl)-4-((4-chlorophenyl)amino)-1,2,5,6-tetrahydropyridine-3-carboxylate (a10)

White solid, M.P.: 211–213 °C; IR (KBr) ν: 3241, 3178, 2976, 2865, 1655, 1592, 1522, 1496, 1350, 1247, 1176, 1067, 810 and 732 cm^−1^.

#### Ethyl 1-(4-bromophenyl)-4-((4-bromophenyl)amino)-2,6-diphenyl-1,2,5,6-tetrahydro-pyridine-3-carboxylate (a11)

White solid, M.P.: 223–227 °C; IR (KBr) ν: 3241, 3087, 2973, 2859, 1646, 1602, 1491, 1372, 1251, 1068, 1011, 800, 719 cm^−1^; ^1^H NMR (250 MHz, CDCl_3_) δ = 10.23 (s, 1H), 7.39–7.07 (m, 14H), 6.39 (d, *J* = 7.3 Hz, 3H), 6.11 (d, *J* = 8.2 Hz, 2H), 5.09 (s, 1H), 4.41 (dqd, *J* = 26.7, 10.7, 7.1, 6.3 Hz, 2H), 2.93–2.63 (m, 2H), 1.46 (t, *J* = 7.1 Hz, 3H).

#### Ethyl 1-(4-bromophenyl)-4-((4-bromophenyl)amino)-2,6-bis(4-chlorophenyl)-1,2,5,6-tetrahydropyridine-3-carboxylate (a12)

White solid, M.P.: 202–205 °C; IR (KBr) ν: 3229, 3068, 2979, 1654, 1590, 1487, 1370, 1250, 1176, 1091, 805, 720 cm^−1^; ^1^H NMR (250 MHz, CDCl_3_) δ = 10.25 (s, 1H), 7.32–7.00 (m, 12H), 6.43–6.18 (m, 5H), 5.05 (s, 1H), 4.58–4.17 (m, 2H), 2.92–2.59 (m, 2H), 1.44 (t, *J* = 7.1 Hz, 3H).

#### Ethyl 1-(4-bromophenyl)-4-((4-bromophenyl)amino)-2,6-bis(3-nitrophenyl)-1,2,5,6-tetra-hydropyridine-3-carboxylate (a13)

White solid, M.P.: 180–183 °C; IR (KBr) ν: 3232, 3068, 2985, 2865, 1662, 1609, 1529, 1349, 1247, 1175, 1068, 805, 731 cm^−1^.

#### Ethyl 2,6-bis(3-bromophenyl)-1-(4-bromophenyl)-4-((4-bromophenyl)amino)-1,2,5,6-tetrahydropyridine-3-carboxylate (a14)

White solid, M.P.: 218–220 °C; IR (KBr) ν: 3226, 3153, 2985, 1647, 1588, 1490, 1369, 1254, 1067, 1011, 807, 785 cm^−1^.

#### Ethyl 1-(4-iodophenyl)-4-((4-iodophenyl)amino)-2,6-diphenyl-1,2,5,6-tetrahydropyridine -3-carboxylate (a15)

White solid, M.P.: 234–237 °C; IR (KBr) ν: 3233, 3059, 2983, 2872, 1648, 1600, 1497, 1317, 1251, 1183, 1073, 796, 698 cm^−1^; ^1^H NMR (250 MHz, CDCl_3_) δ = 10.25 (s, 1H), 7.54–7.27 (m, 11H), 7.25–7.07 (m, 3H), 6.51–6.24 (m, 3H), 5.99 (d, *J* = 8.1 Hz, 2H), 5.12 (s, 1H), 4.59–4.22 (m, 2H), 3.00–2.64 (m, 2H), 1.47 (t, *J* = 7.1 Hz, 3H).


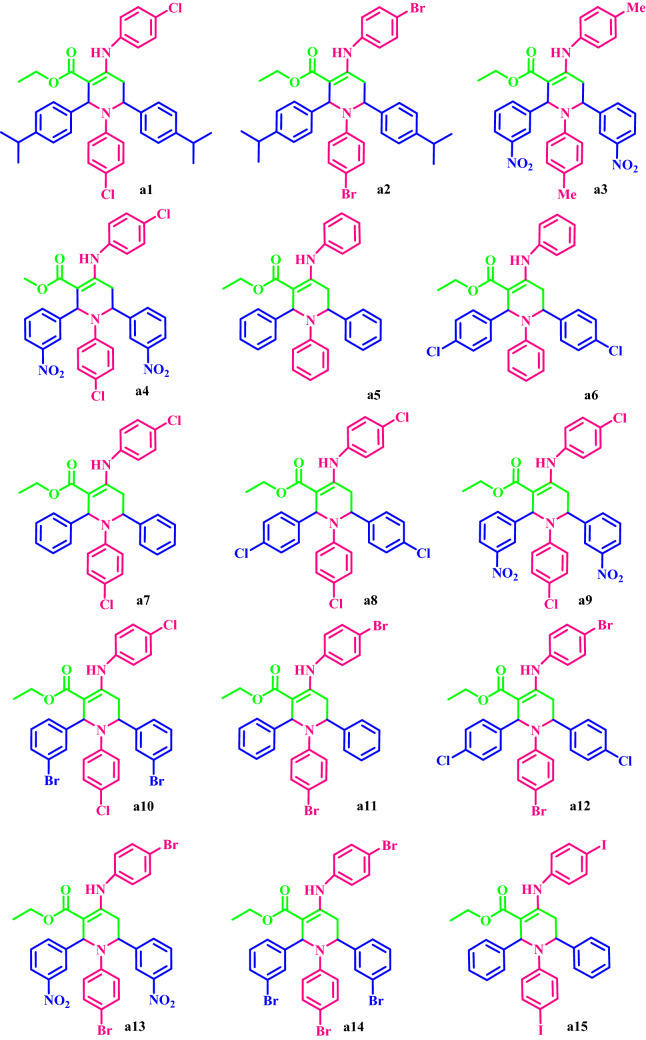


### A proposed mechanism for the synthesis of a(1–15)

**Figure 7:**
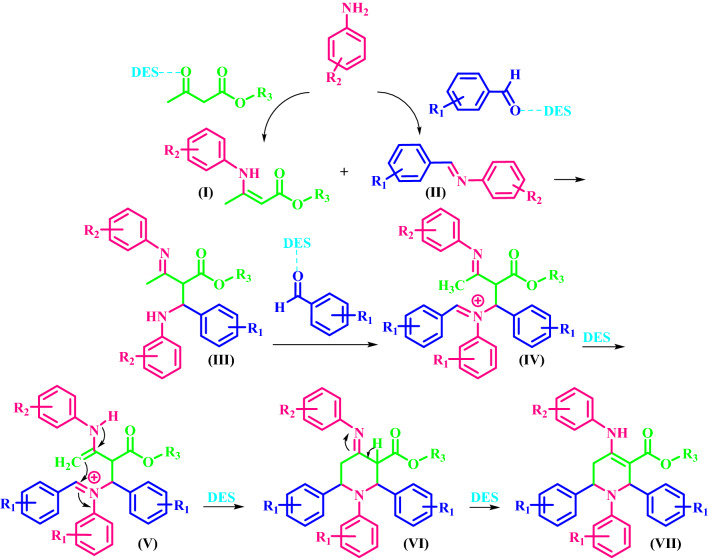
Proposed mechanism for the synthesis of **a(1–15)** by the DES catalyst.

The possible mechanism for the synthesis of **a(1–15)** is shown in Fig. 7. First, the β-keto-ester and aldehyde react with aniline in the presence of DES to give the enamine (**I**) and imine (**II**), respectively. Next, the reaction between the enamine (**I**) and imine (**II**) leads to the intermediate (**III**) via an intermolecular Mannich-type reaction. The intermediate (**III**) reacts with the primary aldehyde to generate intermediate (**IV**). Then, tautomerization of (**IV**) generates intermediate (**V**), which immediately undergoes intramolecular Mannich-type reaction to produce the intermediate (**VI**). In the final step, tautomerization of the intermediate (**VI**) generates the desired product.

### Reusability of ETPP-Br/THF-TCA-DES

**Figure 8:**
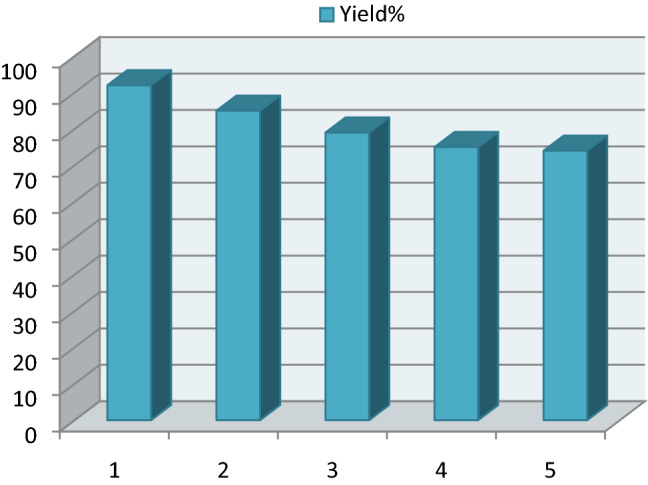
Reusability of ETPP-Br/THF-TCA-DES in the five-consecutive reaction runs.

After completion of the model reaction (synthesis of **a6**) at the optimum point, the reaction was stopped and the mixture was diluted with water and chloroform and shaked. Then, the aqueous layer containing the DES catalyst was separated from the organic layer by simple liquid–liquid extraction, dried to remove water, and reused in consecutive runs without any significant loss of the catalytic activity (92, 85, 79, 75 and 74%, respectively), confirming the stability of the DES catalyst (Fig. 8).

### Comparison of the catalyst activities

Table 6 shows the comparison of different methods for the synthesis of **a(1–15)** indicating the advantage of our proposed procedure over other methods.

**Table 6:** Comparison of the ETPP-Br/THF-TCA-DES catalyst with the other catalysts.

Entry	Catalyst	Condition	Time (h)	Yield (%)	References
1	VitB1	EtOH/H_2_O, reflux	4	90	^44^
2	TMSI	MeOH/rt	24	94	^45^
3	Citric acid	MeOH/rt	8	75	^52^
4	CAN (5 mol %)	MeCN/rt	26	74	^53^
5	2,6-PDCA (10 mol %)	MeOH/rt	7	76	^50^
6	(±)-CSA (10 mol %)	Solvent free, rt	6.5	72	^54^
7	Al(H_2_PO_4_)_3_	EtOH, rt	11	78	^55^
8	Bi(NO_3_)_3_·5H_2_O	EtOH, reflux	19.5	63	^56^
9	Ph_3_CCl (15 mol %)	MeOH, 50 °C	7	72	^57^
10	This work	DES, 80 °C	2.26	80	Present work

### Optimization of the reaction conditions for the synthesis of b(1–10)

The effect of different solvents, temperatures, and amounts of the DES catalyst was investigated on model reaction (the reaction of thioglycolic acid, 4-methylaniline, and 4-chloro-benzaldehyde) to optimize the reaction conditions.

First, the model reaction was performed in ethanol, water, water/ethanol, ethyl acetate, *n*-hexane, and the DES conditions which the DES conditions showed to be the best (Table 7).

**Table 7:** Optimization of solvent by DES.

Entry	Temp. (°C)	Solvent	Yield (%)
1	Reflux	Ethanol	15
2	Reflux	Water	–
3	Reflux	EtOH + H_2_O	55
4	Reflux	Ethyl acetate	45
5	Reflux	*n*-Hexane	–
**6**	**90**	**DES**	**86**

Significant values are in [bold].

Then, the model reaction was conducted in different temperatures which the temperature of 90 °C had the best efficiency (Table 8).

**Table 8:** Optimization of temperature by DES.

Entry	Temp. (°C)	Yield (%)
1	70	65
2	80	77
**3**	**90**	**86**
4	100	72
5	110	90

Significant values are in [bold].

Finally, the model reaction was carried out with different amounts of the DES catalyst which 0.16 mmol of the catalyst had the best yield (Table 9).

**Table 9:** Optimization of the DES amount at 90 °C.

Entry	DES (mmol)	Yield (%)
1	0.5	55
2	0.33	71
**3**	**0.16**	**86**
4	0.12	72
5	0.00	No reaction

Significant values are in [bold].

Overall, we concluded that the best result was found to be the 1.5:1:1 mol ratio of thioglycolic acid, 4-methyl-aniline, and 4-chlorobenzaldehyde with 0.16 mmol of the DES catalyst at 90 °C in appropriate times.

### Synthesis of diverse **b(1–10)**

Based on the results obtained from the optimization conditions (synthesis of **b3**), **b(1–10)** were synthesized from the reaction of thioglycolic acid, aromatic amine, and aldehyde in the presence of ETPP-Br/THF-TCA-DES for an appropriate time (Table 10). Short reaction times and high yields are the important features of the proposed procedure.

**Table 10:** Synthesis of **b(1–10)** by the DES catalyst.


Entry	Aldehyde	Amine	Product	Time (min)	Yield (%)	M.P. °C found, Ref	TON	TOF
1	3-Me-C_7_H_5_O	4-Me-C_6_H_7_N	**b1**	25	83	126–128 NEW	518.7	20.74
2	3-OH-C_7_H_5_O	4-Me-C_6_H_7_N	**b2**	60	80	209–213 NEW	500	8.33
3	4-Cl-C_7_H_5_O	4-Me-C_6_H_7_N	**b3**	40	86	169–170 169–171^58^	537.5	13.43
4	3-NO_2_-C_7_H_5_O	4-Me-C_6_H_7_N	**b4**	30	81	150–152 150–152^58^	506.2	16.8
5	C_7_H_6_O	4-Me-C_6_H_7_N	**b5**	35	75	127–129 136–139^59^	468.7	13.39
6	4-Me-C_7_H_5_O	4-Me-C_6_H_7_N	**b6**	20	85	137–140 129–130^60^	531.2	26.56
7	2,4-diCl-C_7_H_5_O	4-Me-C_6_H_7_N	**b7**	15	90	107–111	562.5	37.5
8	Py-2-aldehyde	4-Me-C_6_H_7_N	**b8**	45	78	159–163 158–162^61^	487.5	10.83
9	C_8_H_6_O_2_	2(4-Me-C_6_H_7_N)	**b9**	90	60	226–228	375	4.16
10	4-Cl-C_7_H_5_O	C_2_N_4_H_4_	**b10**	20	75	216–218	468.7	23.43

### Spectral data^58–61^

#### 2-(*m*-Tolyl)-3-(*p*-tolyl)thiazolidin-4-one (b1)

White solid, M.P.: 126–128 °C; IR (KBr) ν: 3035, 2944, 2859, 1669, 1513, 1374, 1339, 1217, 820, 747 cm^−1^; ^1^H NMR (250 MHz, CDCl_3_) δ = 7.49–6.81 (m, 8H), 6.02 (s, 1H), 3.92 (q, *J* = 15.7 Hz, 2H), 2.29 (s, 3H), 2.25 (s, 3H). ^13^C NMR (62.5 MHz, CDCl_3_) δ 171.04, 138.75, 136.96, 136.61, 134.87, 129.71, 129.50, 126.86, 125.63, 65.52, 33.43, 21.15, 20.99; MW = 283; MS: m/z = 283 [M^+^], 135 (base peak).

#### 2-(3-Hydroxyphenyl)-3-(*p*-tolyl)thiazolidin-4-one (b2)

White solid, M.P.: 209–213 °C; IR (KBr) ν: 3250, 3034, 2905, 2872, 1662, 1602, 1512, 1461, 1387, 1242, 813, 746 cm^−1^; ^1^H NMR (250 MHz, CDCl_3_) δ = 7.25 (d, *J* = 2.6 Hz, 2H), 7.16–7.01 (m, 4H), 6.86–6.68 (m, 2H), 5.97 (s, 1H), 5.15 (s, 1H), 4.07–3.74 (m, 2H), 2.26 (s, 3H). ^13^C NMR (63 MHz, CDCl_3_) δ 171.10, 149.99, 130.08, 129.75, 125.43, 119.21, 115.96, 113.49, 65.40, 33.29, 20.94; MW = 285; MS: m/z = 285 [M^+^], 137 (base peak).

#### 2-(4-Chlorophenyl)-3-(*p*-tolyl)thiazolidin-4-one (b3)

White solid, M.P.: 169–170 °C; IR (KBr) ν: 3057, 3031, 2939, 1669, 1513, 1378, 1219, 805, 749 cm^−1^; ^1^H NMR (250 MHz, CDCl_3_) δ = 7.24 (s, 4H), 7.04 (q, *J* = 8.0 Hz, 4H), 6.03 (s, 1H), 4.08–3.76 (m, 2H), 2.25 (s, 3H).

#### 2-(3-Nitrophenyl)-3-(*p*-tolyl)thiazolidin-4-one (b4)

Yellow solid, M.P.: 150–152 °C; IR (KBr) ν: 3050, 3021, 2922, 1670, 1530, 1391, 1351, 1219, 807, 725 cm^−1^; ^1^H NMR (250 MHz, CDCl_3_) δ = 8.26–7.98 (m, 1H), 7.75–7.37 (m, 1H), 7.19–6.89 (m, 1H), 6.16 (s, 0H), 4.17–3.79 (m, 1H), 2.25 (s, 1H).

#### 2-Phenyl-3-(*p*-tolyl)thiazolidin-4-one (b5)

White solid, M.P.: 127–129 °C; IR (KBr) ν: 3034, 2938, 1669, 1379, 1348, 1216, 726 cm^−1^.

#### 2,3-Di-*p*-tolylthiazolidin-4-one (b6)

White solid, M.P.: 137–140 °C; IR (KBr) ν: 3037, 2975, 2922, 2899, 2852, 1697, 1512, 1354, 809, 740 cm^−1^.

#### 2-(2,4-Dichlorophenyl)-3-(*p*-tolyl)thiazolidin-4-one (b7)

White solid, M.P.: 107–111 °C; IR (KBr) ν: 3030, 2971, 2925, 2859, 1690, 1587, 1510, 1364, 818, 501 cm^−1^.

#### 2-(Pyridin-2-yl)-3-(*p*-tolyl)thiazolidin-4-one (b8)

Yellow solid, M.P.: 159–163 °C; IR (KBr) ν: 3030, 2964, 2912, 1670, 1590, 1514, 1392, 1329, 1217, 748 cm^−1^.

#### 2,2'-(1,4-Phenylene)bis(3-(*p*-tolyl)thiazolidin-4-one) (b9)

White solid, M.P.: 226–228 °C; IR (KBr) ν: 3037, 2971, 2928, 1666, 1513, 1379, 1221, 818, 737 cm^−1^.

#### 2-(4-Chlorophenyl)-3-(1H-1,2,4-triazol-5-yl)thiazolidin-4-one (b10)

White solid, M.P.: 216–218 °C; IR (KBr) ν: 3214, 3118, 2984, 1606, 1520, 1464, 1379, 1333, 833, 772 cm^−1^.


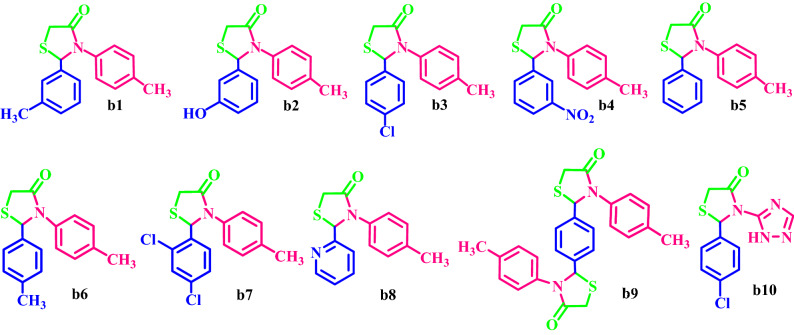


### A proposed mechanism for the synthesis of b(1–10)

**Figure 9:**
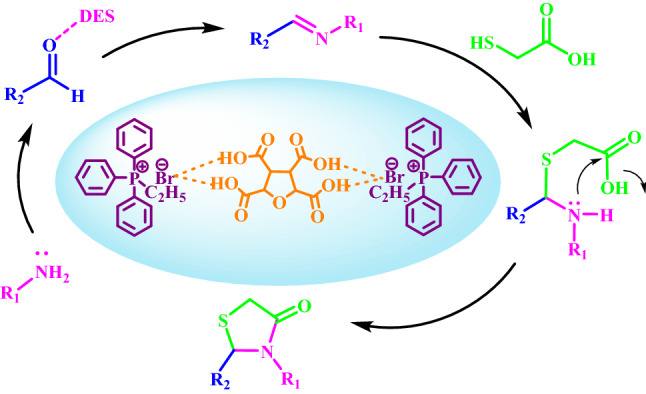
Proposed mechanism for the synthesis of **b(1–10)**.

When the carbonyl oxygen is coordinated to DES, it is activated to be attacked by the amine to form imine. Then, the nucleophilic attack of the sulfur moiety of thioglycolic acid on the imine carbon followed by the intramolecular cyclization and the subsequent removing of H_2_O, giving rise to the cyclized thiazolidinone product (Fig. 9).

### Reusability of the ETPP-Br/THF-TCA-DES catalyst

**Figure 10:**
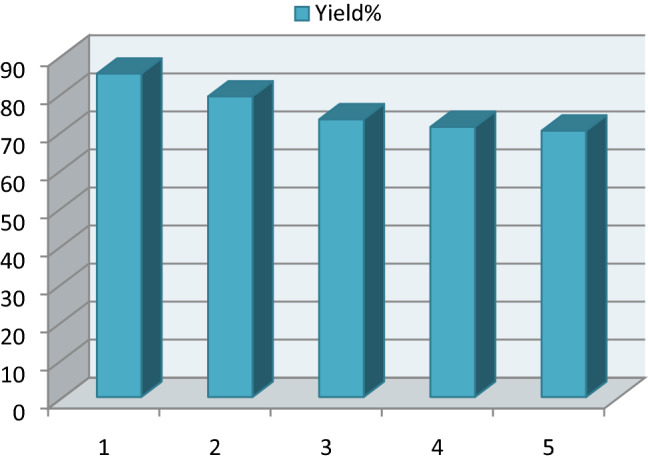
Reusability of the DES catalyst in five-consecutive reaction runs.

After completion of the model reaction (synthesis of **b3**) at the optimum point, the reaction was stopped and the mixture was diluted with water and ethyl acetate and shaked. The DES catalyst inside the aqueous layer was separated from the organic layer by simple liquid–liquid extraction and dried to remove water. Then, it was reused in consecutive runs which showed a small decrease in the catalytic activity (85, 79, 73, 71 and 70%, respectively), confirming the stability of the DES catalyst (Fig. 10).

### Comparison of the ETPP-Br/THF-TCA-DES catalyst activity with other previous reports

Table 11 compares the catalytic activity of the DES catalyst with other previous methods reported in the literature for the synthesis of **b(1–10)**.

**Table 11:** Comparison of the ETPP-Br-DES catalyst with the other catalysts.

Entry	Catalyst	Condition	Time (h)	Yield (%)	References
1	Protic acid	PhMe/100 °C	4.4	78	^62^
2	Nano-CdZr_4_(PO_4_)_6_	PhMe/ultrasonic	28 min	83	^63^
3	Silica gel	CH_2_Cl_2_	5	87	^64^
4	L_3_·CuSO_4_·5H_2_O	PhMe/110 °C	12	88	^65^
5	Bi(SCH_2_COOH)_3_	Solvent free/70 °C	2	80	^66^
6	APS	Solvent free/90 °C	12	83	^67^
7	PEG-400	Solvent free/50 °C	2	78	^68^
8	Ionic liquid	120 °C	3	88	^69^
9	VOSO_4_	MeCN/ultrasonic	50 min	82	^70^
10	This work	DES, 90 °C	38 min	80	Present work

## Conclusion

In conclusion, a novel Deep Eutectic Solvent was prepared by a mixture (mole ratio 7:3) of ETPP-Br and THF-TCA, and used as a capable and new catalyst for the synthesis of:

1. the four new **[a(1–4)]** and the eleven **[a(5–15)]** known alkyl 1,2,6-trisubstituted-4-[(hetero) arylamino]-1,2,5,6-tetrahydropyridine-3-carboxylates, and
2. the two new **[b(1–2)]** and the eight **[b(3–10)]** known 1,3-thiazolidin-4-ones in DES conditions, short reaction time, high yields, and easy recycling and separation of the DES catalyst.

Also, formation of a novel DES was confirmed by the FT-IR, the ^1^H NMR and the eutectic point curve, and there is a very nice consistency between them. The ratio of the peak integration values of Hs of the CH_3_ groups, Hs of the CH_2_ groups, Hs of the phenyl groups, Hs of the tetra acid, and Hs of the THF cycle is 6:4:30:4:4 indicating the presence of the two C_2_H_5_ groups, the six phenyl groups, the four acidic hydrogens, and the one THF cycle.

Incidentally, the decrease in splitting patterns of the peaks in DES, compared to the two starting materials can be very nice evidence of hydrogen bond formation between the two components.

## Supplementary Information

Supplementary Information.

## Data Availability

All data generated or analyzed during this study are included in this published article and its supplementary information files.
