# Supplementary material for: Application of a novel deep eutectic solvent as a capable and new catalyst for the synthesis of tetrahydropyridines and 1,3-thiazolidin-4-ones
Source: Sci Rep. 2023 Apr 10;13:5804. doi: 10.1038/s41598-023-32882-0 (PMC10086034; doi:10.1038/s41598-023-32882-0)
Supplement: Supplementary file 1 — Supplementary Information. [file 41598_2023_32882_MOESM1_ESM.docx]

**Supporting Information**

**Scientific Reports**

**Application of a novel Deep Eutectic Solvent as a capable and new catalyst for the synthesis of tetrahydropyridines and 1,3-thiazolidin-4-ones**

**Hadis Goudarzi, Davood Habibi*, Arezo Monem**

Department of Organic Chemistry, Faculty of Chemistry, Bu-Ali Sina University, Hamedan 6517838683, Iran

*****Corresponding author email: davood.habibi@gmail.com, Tel: +98 81 38380922; Fax: +98 81 38380709

**Content Page**

^1^H NMR spectrum of ethyl triphenylphosphonium-bromide (ETPP-Br) 3

^1^H NMR spectrum of tetrahydrofuran-2,3,4,5-tetracarboxylic acid (THF-TCA) 3

FT-IR spectrum of a1 4

^1^H NMR spectrum of a1 4

^13^C NMR spectrum of a1 5

Mass spectrum of a1 5

FT-IR spectrum of a2 6

^1^H NMR spectrum of a2 6

^13^C NMR spectrum of a2 7

Mass spectrum of a2 7

FT-IR spectrum of a3 8

^1^H NMR spectrum of a3 8

^13^C NMR spectrum of a3 9

Mass spectrum of a3 9

FT-IR spectrum of a4 10

^1^H NMR spectrum of a4 10

^13^C NMR spectrum of a4 11

Mass spectrum of a4 11

FT-IR spectrum of a5 12

^1^H NMR spectrum of a5 12

FT-IR spectrum of a6 13

^1^H NMR spectrum of a6 13

FT-IR spectrum of a7 14

^1^H NMR spectrum of a7 14

FT-IR spectrum of a8 15

^1^H NMR spectrum of a8 15

FT-IR spectrum of a9 16

^1^H NMR spectrum of a9 16

FT-IR spectrum of a10 17

FT-IR spectrum of a11 17

^1^H NMR spectrum of a11 18

FT-IR spectrum of a12 18

^1^H NMR spectrum of a12 19

FT-IR spectrum of a13 19

FT-IR spectrum of a14 20

FT-IR spectrum of a15 20

^1^H NMR spectrum of a15 21

FT-IR spectrum of b1 21

^1^H NMR spectrum of b1 22

^13^C NMR spectrum of b1 22

Mass spectrum of b1 23

FT-IR spectrum of b2 23

^1^H NMR spectrum of b2 24

^13^C NMR spectrum of b2 24

Mass spectrum of b2 25

FT-IR spectrum of b3 25

^1^H NMR spectrum of b3 26

FT-IR spectrum of b4 26

^1^H NMR spectrum of b4 27

FT-IR spectrum of b5 27

FT-IR spectrum of b6 28

FT-IR spectrum of b7 28

FT-IR spectrum of b8 28

FT-IR spectrum of b9 29

FT-IR spectrum of b10 29

Experimental (reagents, solvents, chemicals, the scientific devices) 29

^1^H NMR spectrum of ethyl triphenylphosphonium-bromide (ETPP-Br):

^1^H NMR spectrum of tetrahydrofuran-2,3,4,5-tetracarboxylic acid (THF-TCA):

FT-IR spectrum of Ethyl 1-(4-chlorophenyl)-4-((4-chlorophenyl)amino)-2,6-bis(4-isopropylphenyl)-1,2,5,6 tetrahydropyridine-3-carboxylate (a1):


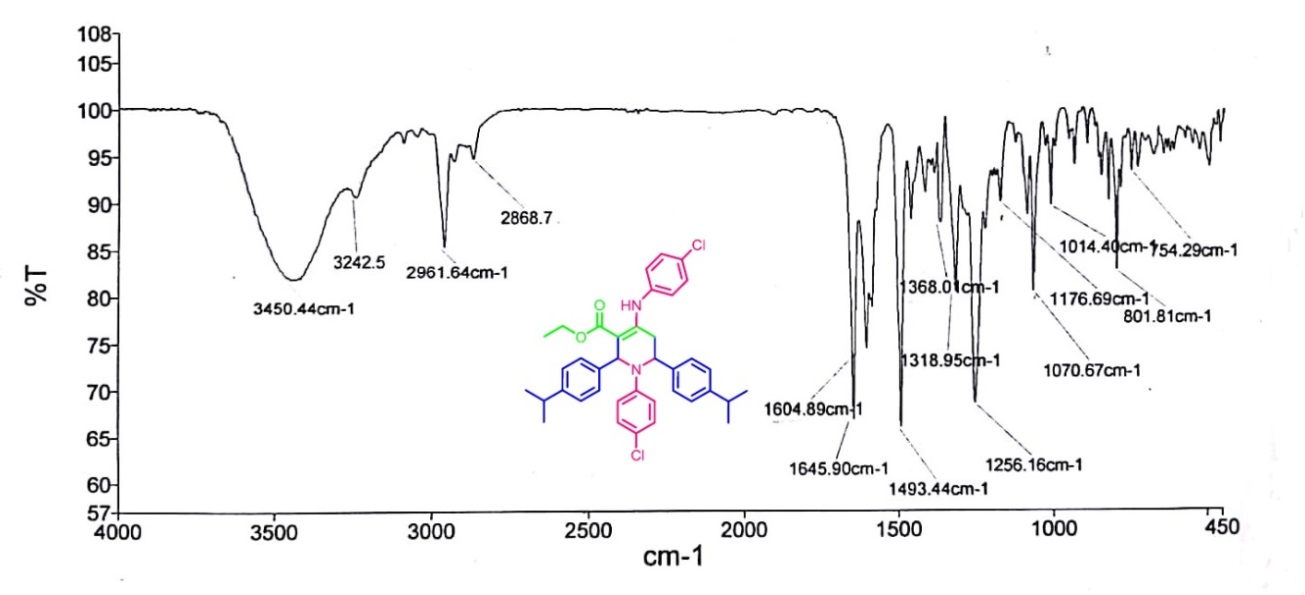


^1^HNMR spectrum of Ethyl 1-(4-chlorophenyl)-4-((4-chlorophenyl)amino)-2,6-bis(4-isopropylphenyl)-1,2,5,6 tetrahydropyridine-3-carboxylate (a1):


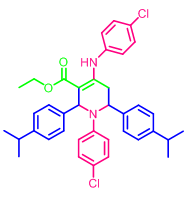


^13^CNMR spectrum of Ethyl 1-(4-chlorophenyl)-4-((4-chlorophenyl)amino)-2,6-bis(4-isopropylphenyl)-1,2,5,6 tetrahydropyridine-3-carboxylate (a1):


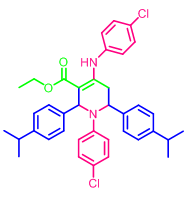

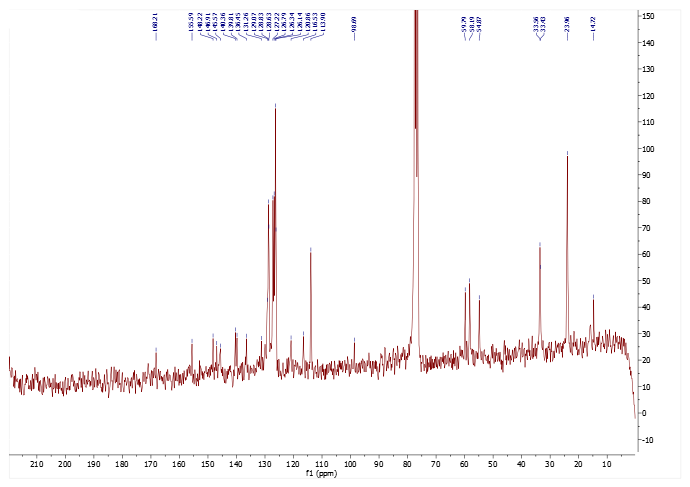


Mass spectrum of Ethyl 1-(4-chlorophenyl)-4-((4-chlorophenyl)amino)-2,6-bis(4-isopropylphenyl)-1,2,5,6 tetrahydropyridine-3-carboxylate (a1):


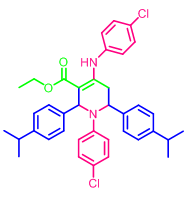

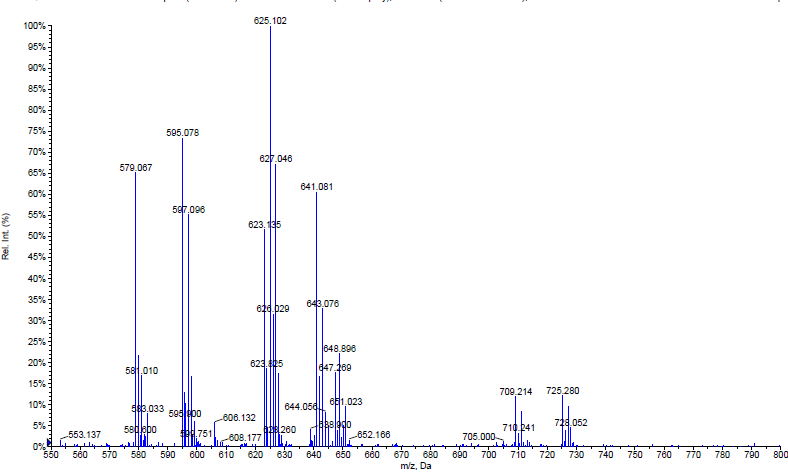


FT-IR spectrum of Ethyl 1-(4-bromophenyl)-4-((4-bromophenyl)amino)-2,6-bis(4-isopropylphenyl)-1,2,5,6-tetrahydropyridine-3-carboxylate (a2):


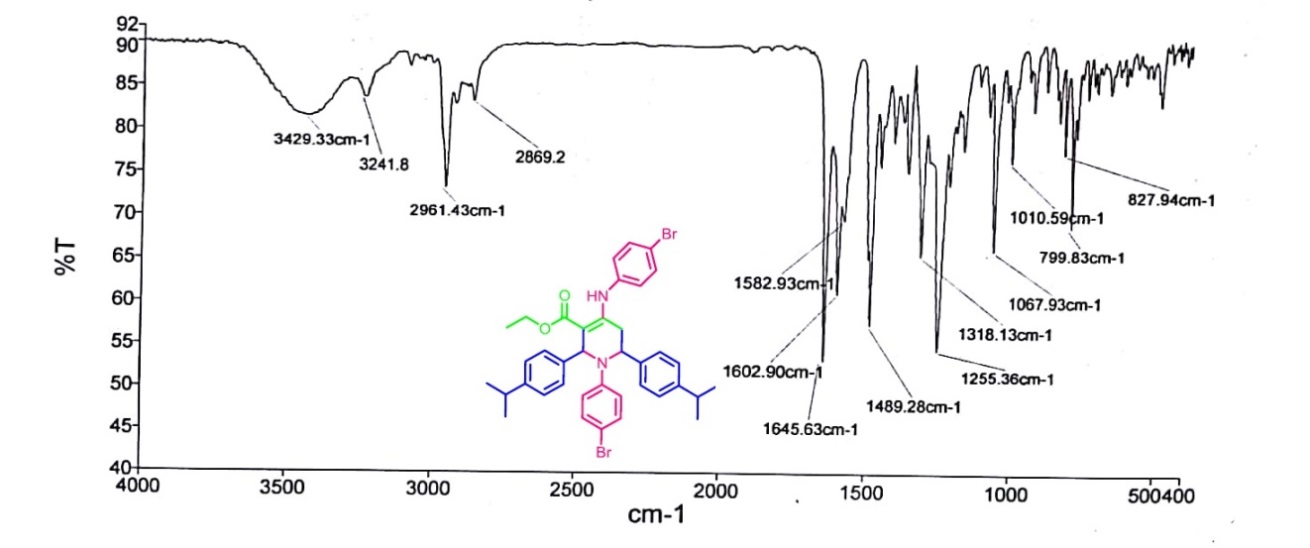


^1^HNMR spectrum of Ethyl 1-(4-bromophenyl)-4-((4-bromophenyl)amino)-2,6-bis(4-isopropylphenyl)-1,2,5,6-tetrahydropyridine-3-carboxylate (a2):


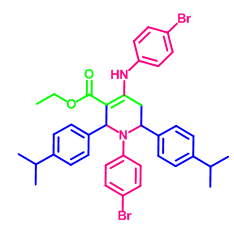


^13^CNMR spectrum of Ethyl 1-(4-bromophenyl)-4-((4-bromophenyl)amino)-2,6-bis(4-isopropylphenyl)-1,2,5,6-tetrahydropyridine-3-carboxylate (a2):


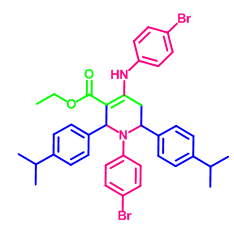

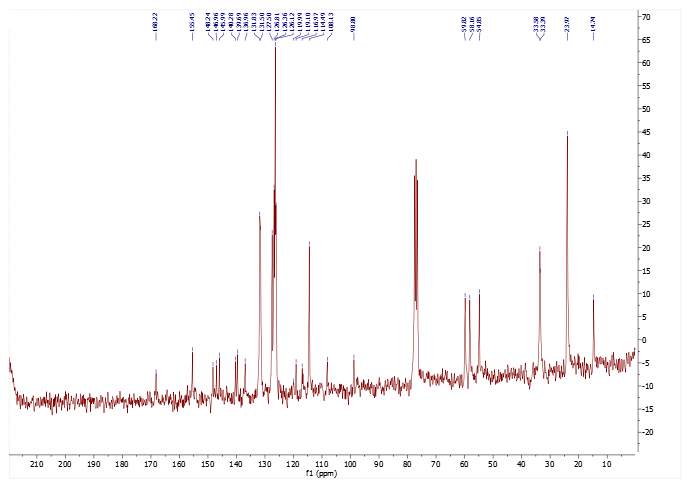


Mass spectrum of Ethyl 1-(4-bromophenyl)-4-((4-bromophenyl)amino)-2,6-bis(4-isopropylphenyl)-1,2,5,6-tetrahydropyridine-3-carboxylate (a2):


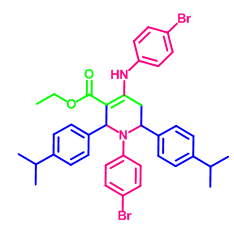

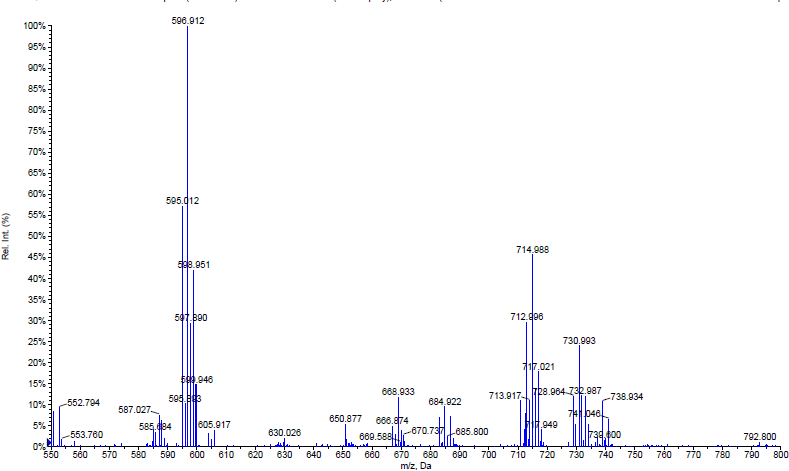


FT-IR spectrum of Ethyl 2,6-bis(3-nitrophenyl)-1-(p-tolyl)-4-(p-tolylamino)-1,2,5,6-tetrahydropyridine-3-carboxylate (a3):


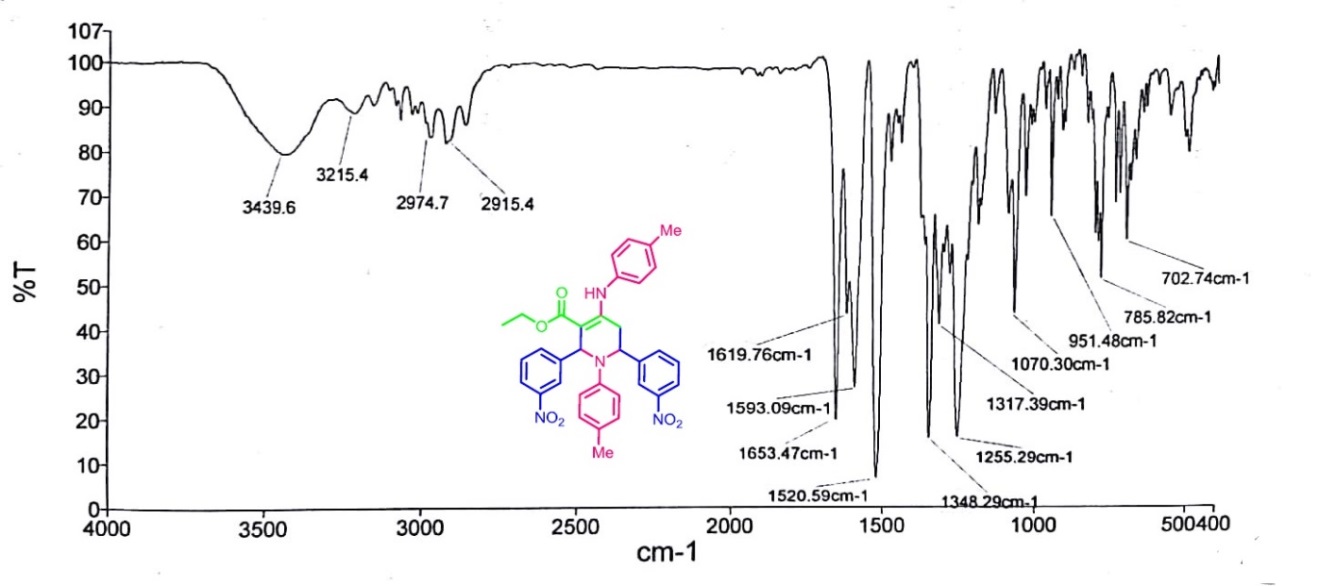


^1^HNMR spectrum of Ethyl 2,6-bis(3-nitrophenyl)-1-(p-tolyl)-4-(p-tolylamino)-1,2,5,6-tetrahydropyridine-3-carboxylate (a3):


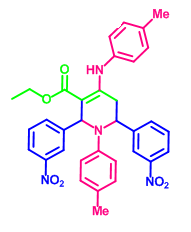


^13^CNMR spectrum of Ethyl 2,6-bis(3-nitrophenyl)-1-(p-tolyl)-4-(p-tolylamino)-1,2,5,6-tetrahydropyridine-3-carboxylate (a3):


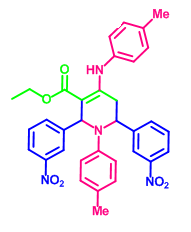

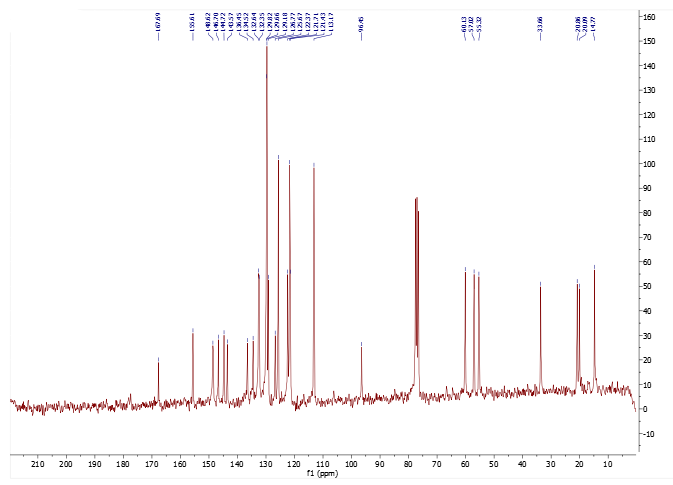


Mass spectrum of Ethyl 2,6-bis(3-nitrophenyl)-1-(p-tolyl)-4-(p-tolylamino)-1,2,5,6-tetrahydropyridine-3-carboxylate (a3):


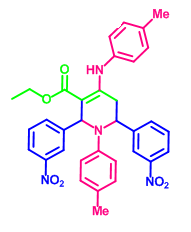

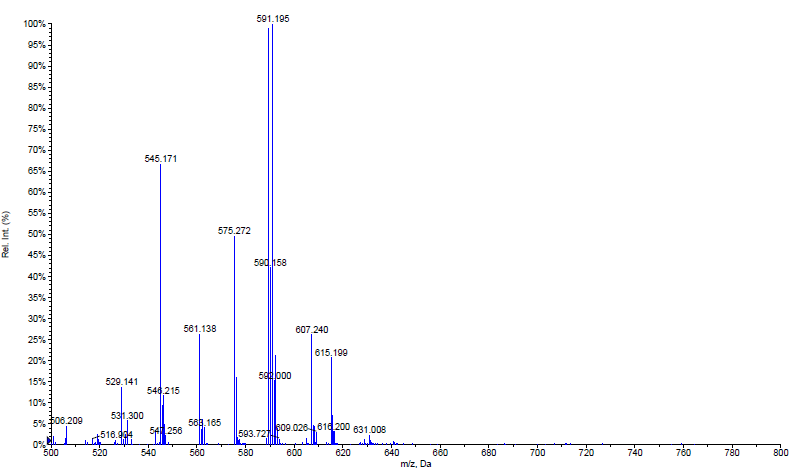


FT-IR spectrum of Methyl 1-(4-chlorophenyl)-4-((4-chlorophenyl)amino)-2,6-bis(3-nitrophenyl)-1,2,5,6-tetrahydropyridine-3-carboxylate (a4):


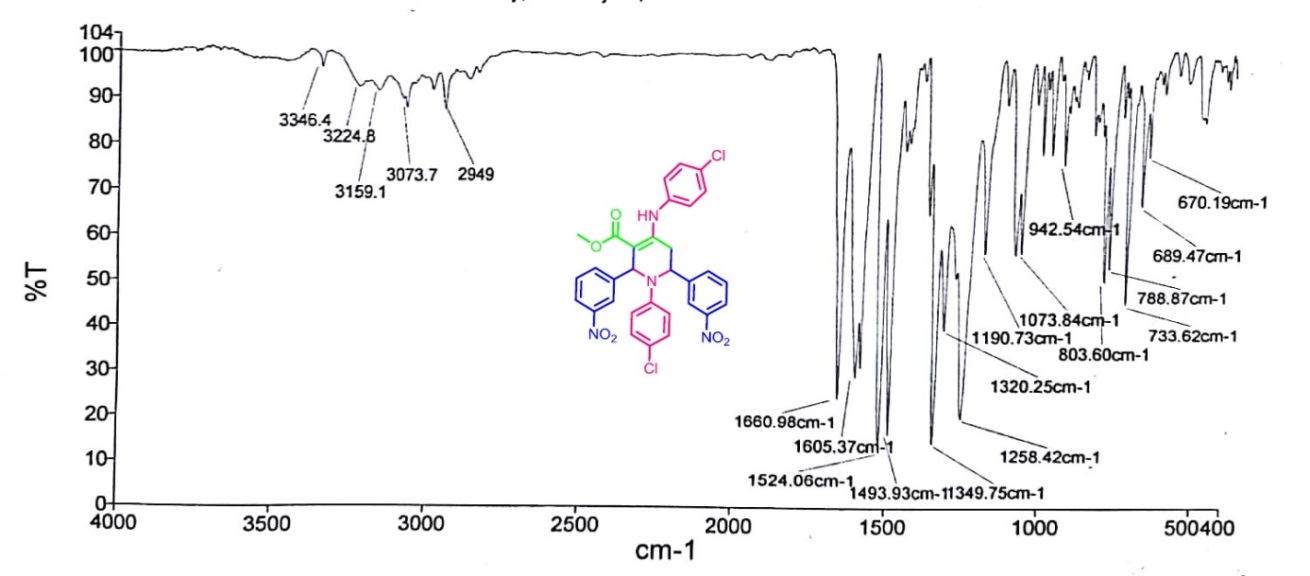


^1^HNMR spectrum of Methyl 1-(4-chlorophenyl)-4-((4-chlorophenyl)amino)-2,6-bis(3-nitrophenyl)-1,2,5,6-tetrahydropyridine-3-carboxylate (a4):


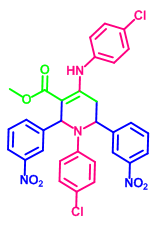


^13^CNMR spectrum of Methyl 1-(4-chlorophenyl)-4-((4-chlorophenyl)amino)-2,6-bis(3-nitrophenyl)-1,2,5,6-tetrahydropyridine-3-carboxylate (a4):


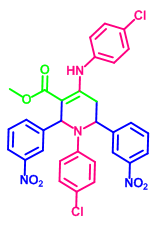

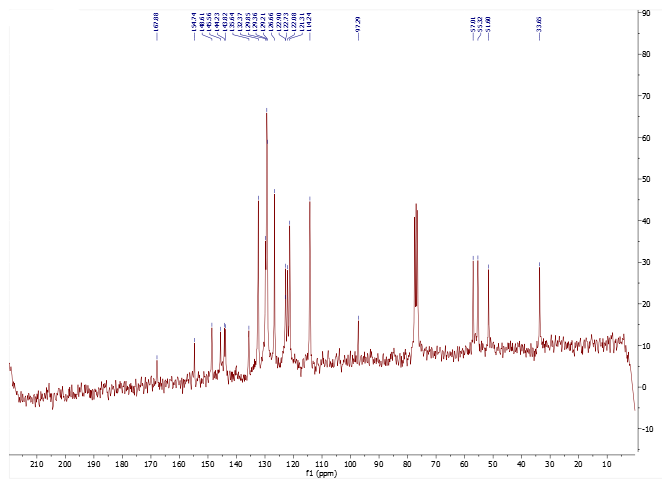


Mass spectrum of Methyl 1-(4-chlorophenyl)-4-((4-chlorophenyl)amino)-2,6-bis(3-nitrophenyl)-1,2,5,6-tetrahydropyridine-3-carboxylate (a4):


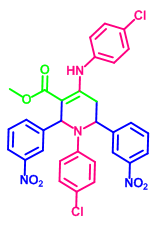

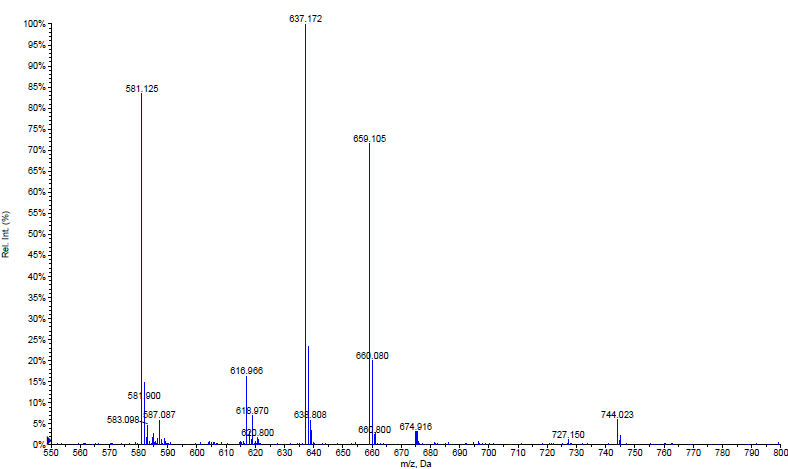


FT-IR spectrum of Ethyl 1,2,6-triphenyl-4-(phenylamino)-1,2,5,6-tetrahydropyridine-3-carboxylate (a5):


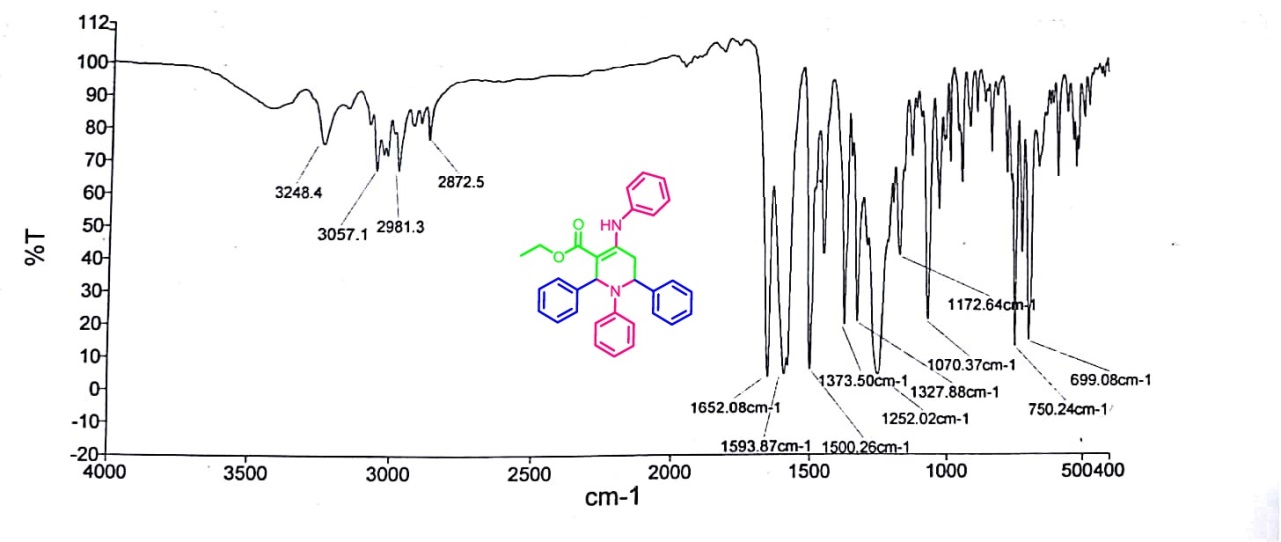


^1^HNMR spectrum of Ethyl 1,2,6-triphenyl-4-(phenylamino)-1,2,5,6-tetrahydropyridine-3-carboxylate (a5):


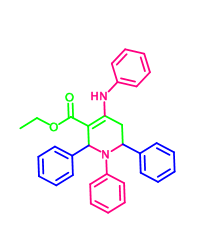


FT-IR spectrum of Ethyl 2,6-bis(4-chlorophenyl)-1-phenyl-4-(phenylamino)-1,2,5,6-tetrahydropyridine-3-carboxylate (a6):


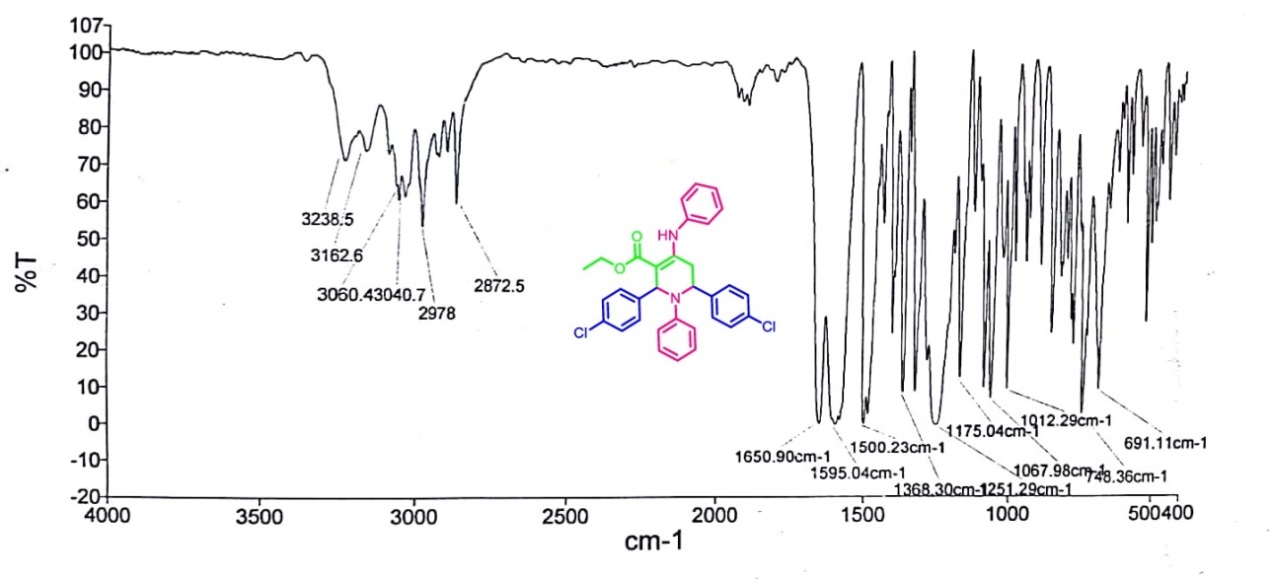


^1^HNMR spectrum of Ethyl 2,6-bis(4-chlorophenyl)-1-phenyl-4-(phenylamino)-1,2,5,6-tetrahydropyridine-3-carboxylate (a6):


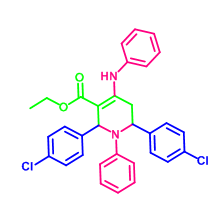


FT-IR spectrum of Ethyl 1-(4-chlorophenyl)-4-((4-chlorophenyl)amino)-2,6-diphenyl-1,2,5,6-tetrahydro-pyridine-3-carboxylate (a7):


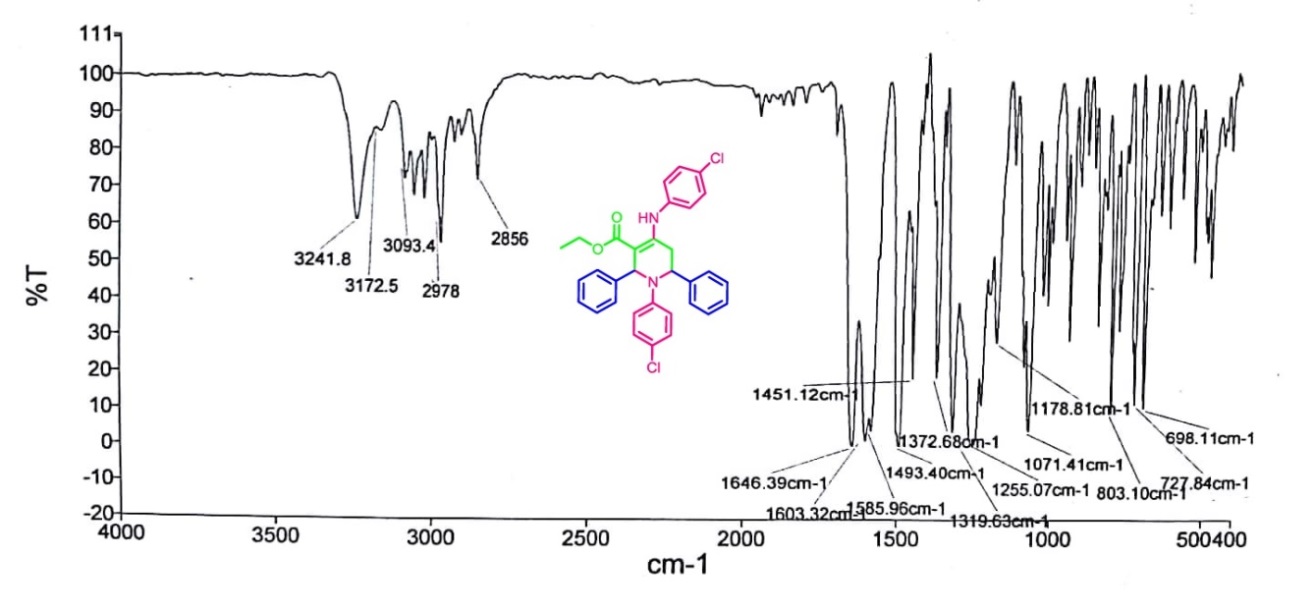


^1^HNMR spectrum of Ethyl 1-(4-chlorophenyl)-4-((4-chlorophenyl)amino)-2,6-diphenyl-1,2,5,6-tetrahydro-pyridine-3-carboxylate (a7):


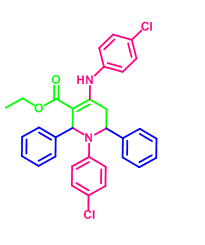


FT-IR spectrum of Ethyl 1,2,6-tris(4-chlorophenyl)-4-((4-chlorophenyl)amino)-1,2,5,6-tetrahydropyridine-3-carboxylate (a8):


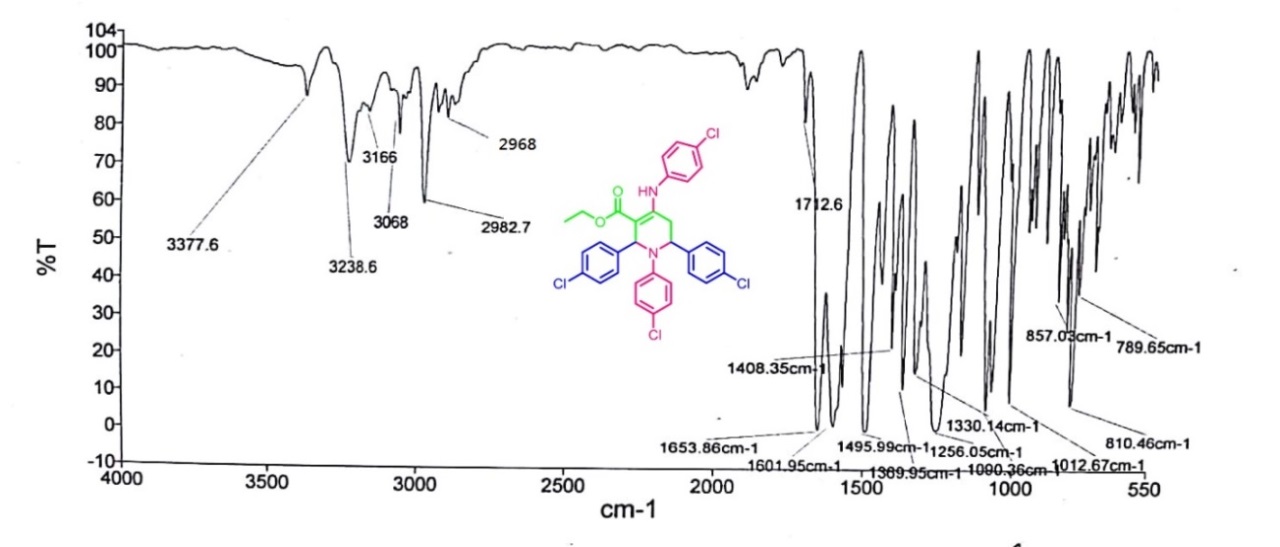


^1^HNMR spectrum of Ethyl 1,2,6-tris(4-chlorophenyl)-4-((4-chlorophenyl)amino)-1,2,5,6-tetrahydropyridine-3-carboxylate (a8):


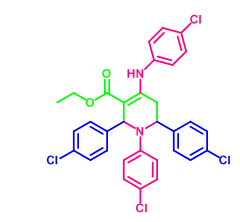


FT-IR spectrum of Ethyl 1-(4-chlorophenyl)-4-((4-chlorophenyl)amino)-2,6-bis(3-nitrophenyl)-1,2,5,6-tetrahydropyridine-3-carboxylate (a9):


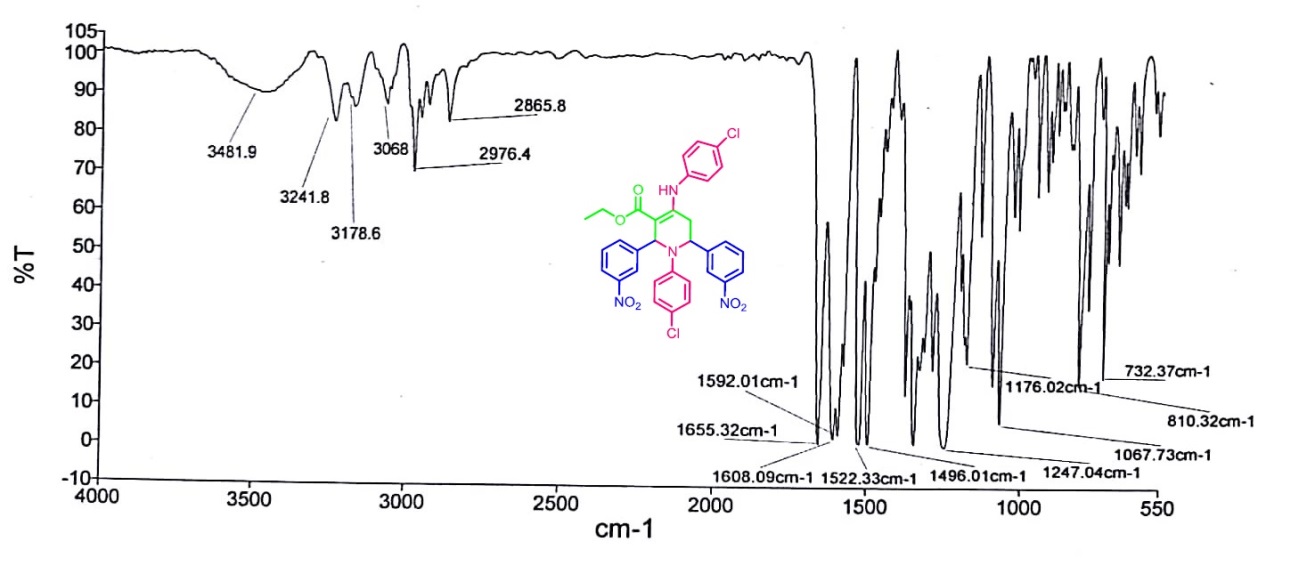


^1^HNMR spectrum of Ethyl 1-(4-chlorophenyl)-4-((4-chlorophenyl)amino)-2,6-bis(3-nitrophenyl)-1,2,5,6-tetrahydropyridine-3-carboxylate (a9):


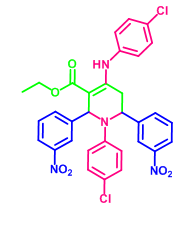


FT-IR spectrum of Ethyl 2,6-bis(3-bromophenyl)-1-(4-bromophenyl)-4-((4-bromophenyl)amino)-1,2,5,6-tetrahydropyridine-3-carboxylate (a10):


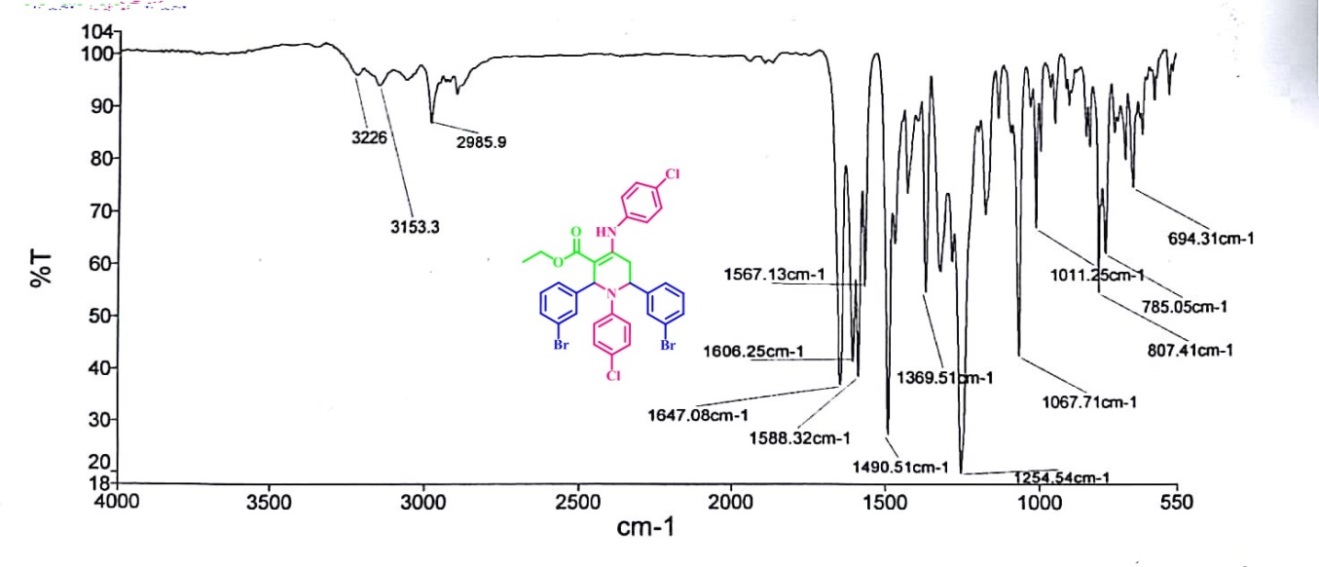


FT-IR spectrum of Ethyl 1-(4-bromophenyl)-4-((4-bromophenyl)amino)-2,6-diphenyl-1,2,5,6-tetrahydro-pyridine-3-carboxylate (a11):


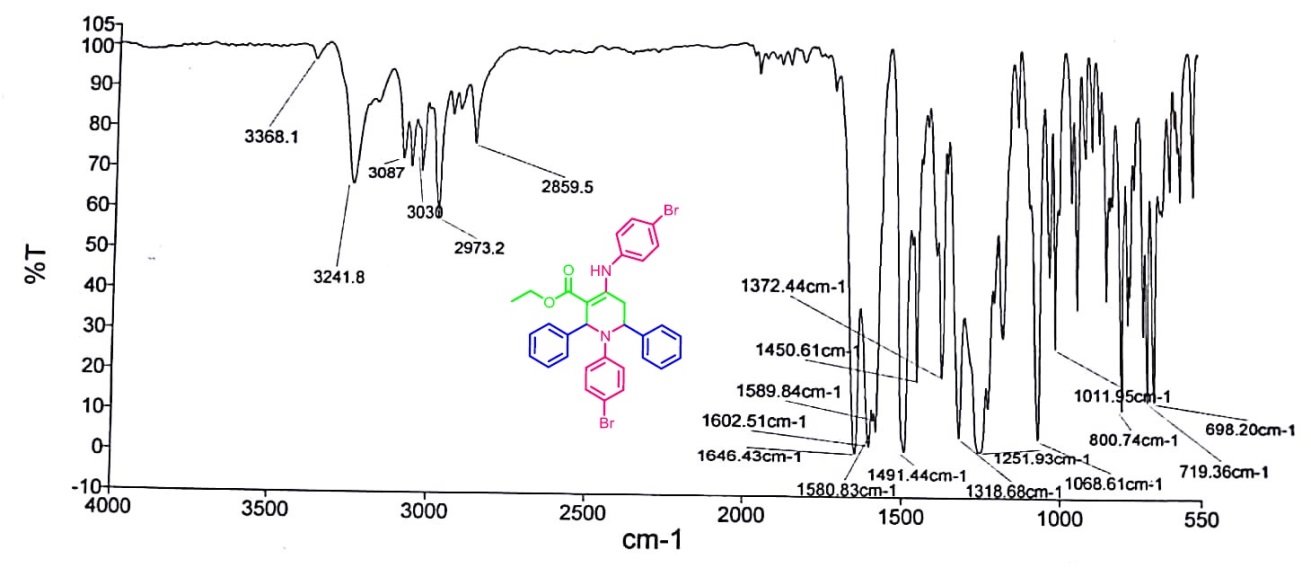


^1^HNMR spectrum of Ethyl 1-(4-bromophenyl)-4-((4-bromophenyl)amino)-2,6-diphenyl-1,2,5,6-tetrahydro-pyridine-3-carboxylate (a11):


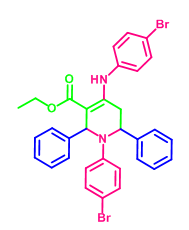


FT-IR spectrum of Ethyl 1-(4-bromophenyl)-4-((4-bromophenyl)amino)-2,6-bis(4-chlorophenyl)-1,2,5,6-tetrahydropyridine-3-carboxylate (a12):


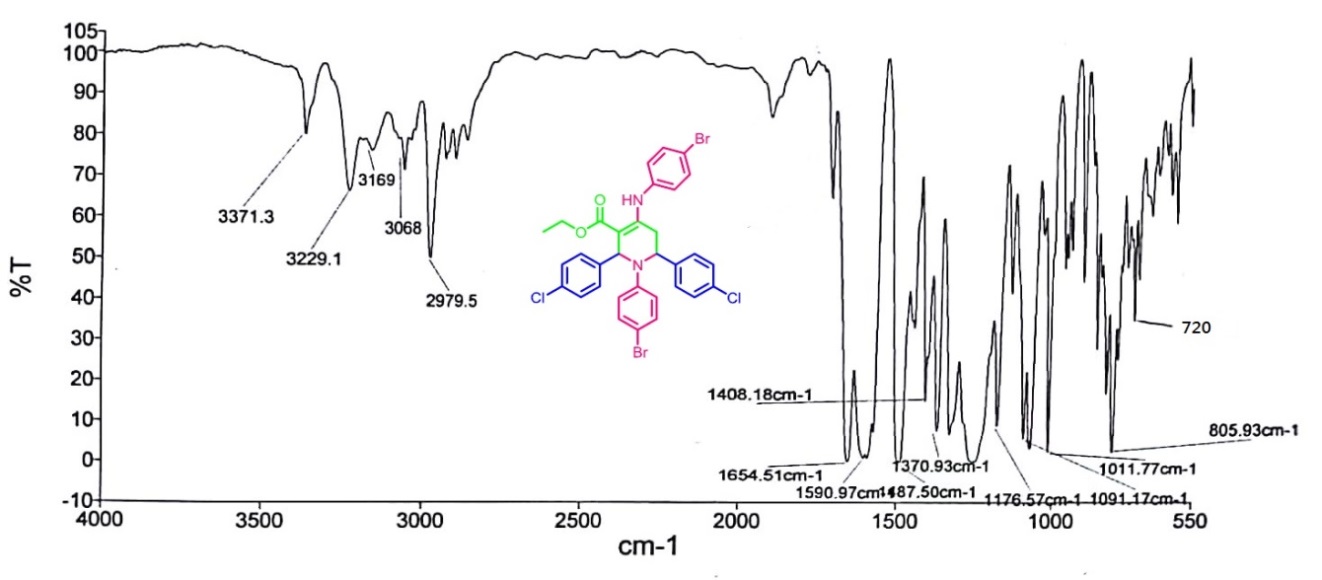


^1^HNMR spectrum of Ethyl 1-(4-bromophenyl)-4-((4-bromophenyl)amino)-2,6-bis(4-chlorophenyl)-1,2,5,6-tetrahydropyridine-3-carboxylate (a12):


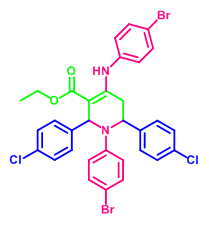


FT-IR spectrum of Ethyl 1-(4-bromophenyl)-4-((4-bromophenyl)amino)-2,6-bis(3-nitrophenyl)-1,2,5,6-tetrahydropyridine-3-carboxylate (a13):


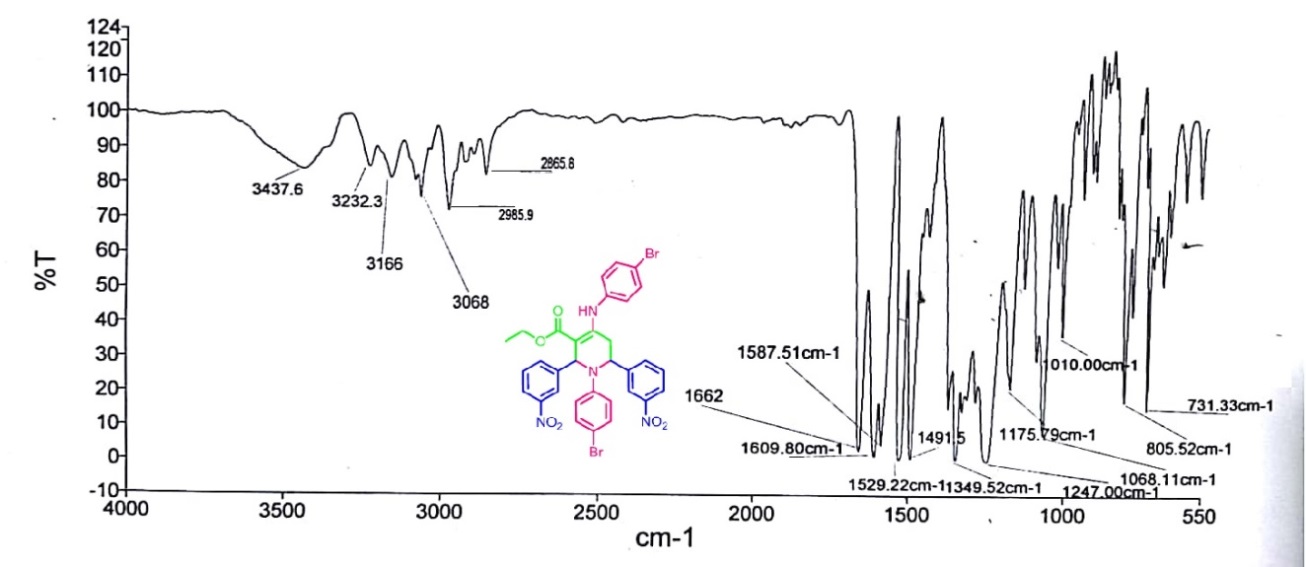


FT-IR spectrum of Ethyl 2,6-bis(3-bromophenyl)-1-(4-bromophenyl)-4-((4-bromophenyl)amino)-1,2,5,6-tetrahydropyridine-3-carboxylate (a14):


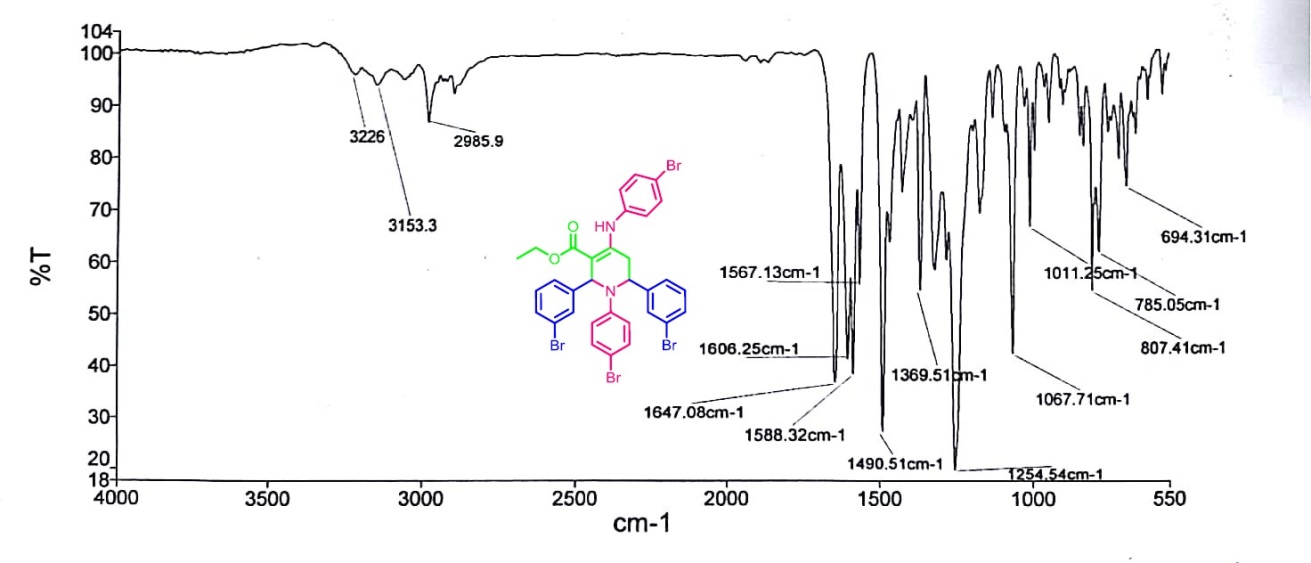


FT-IR spectrum of Ethyl 1-(4-iodophenyl)-4-((4-iodophenyl)amino)-2,6-diphenyl-1,2,5,6-tetrahydropyridine-3-carboxylate (a15):


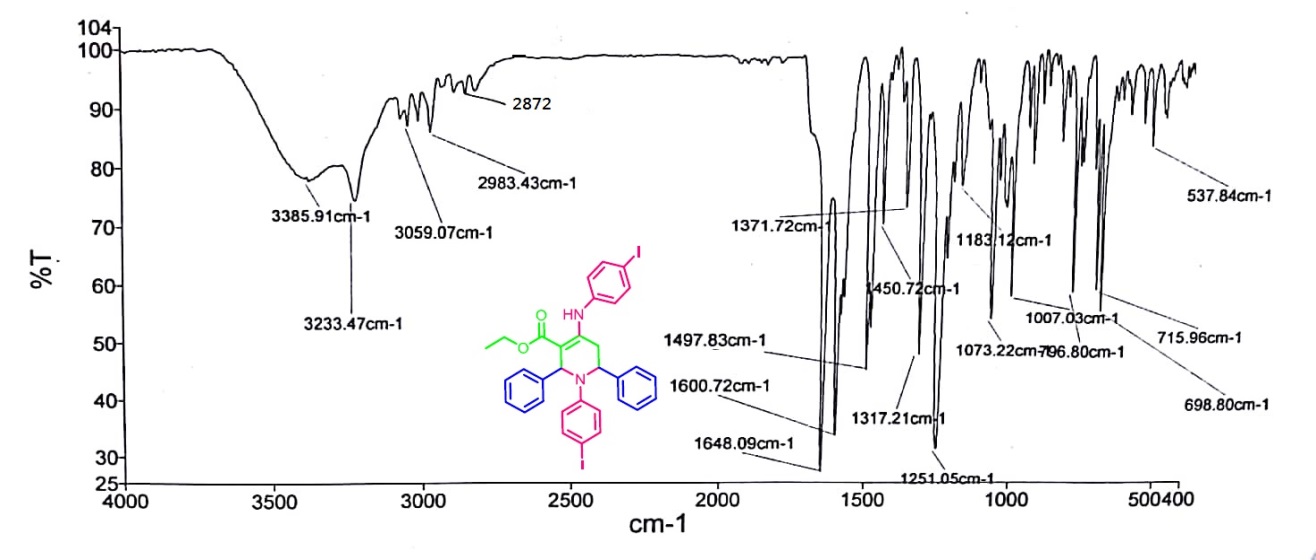


^1^HNMR spectrum of Ethyl 1-(4-iodophenyl)-4-((4-iodophenyl)amino)-2,6-diphenyl-1,2,5,6-tetrahydropyridine-3-carboxylate (a15):


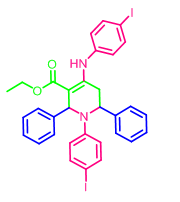


FT-IR spectrum of 2-(m-Tolyl)-3-(p-tolyl)thiazolidin-4-one (b1):


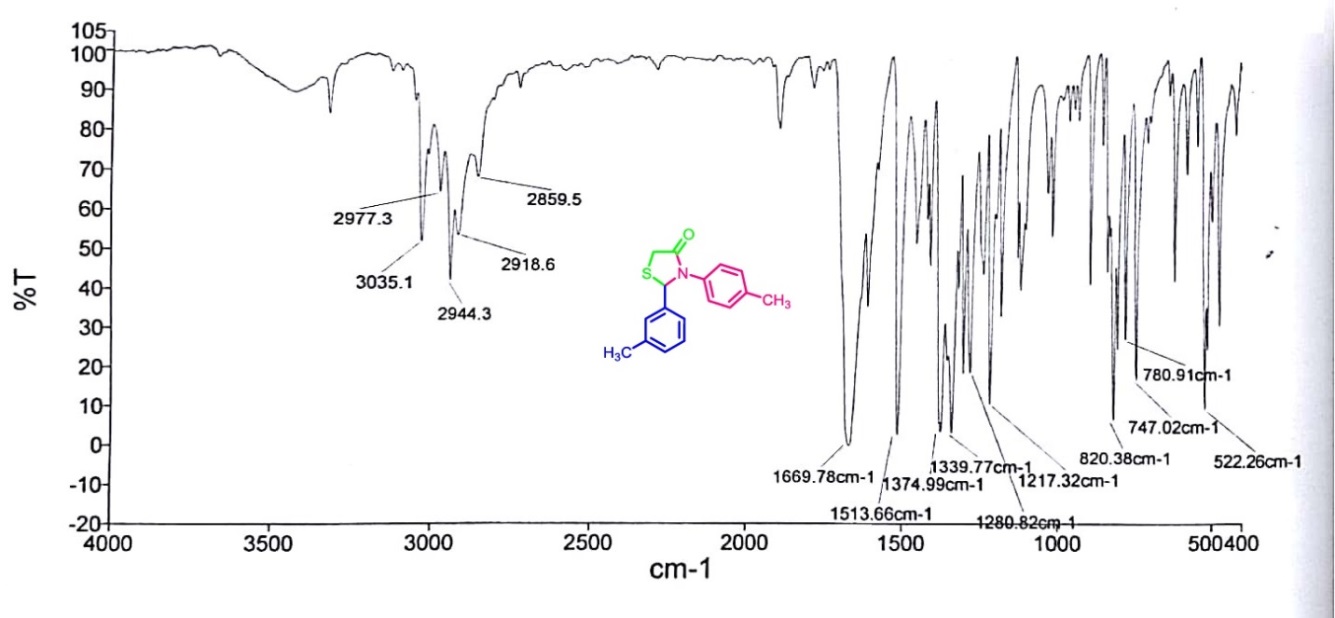


^1^HNMR spectrum of 2-(m-Tolyl)-3-(p-tolyl)thiazolidin-4-one (b1):


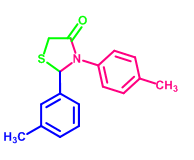


^13^CNMR spectrum of 2-(m-Tolyl)-3-(p-tolyl)thiazolidin-4-one (b1):


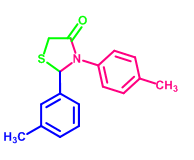

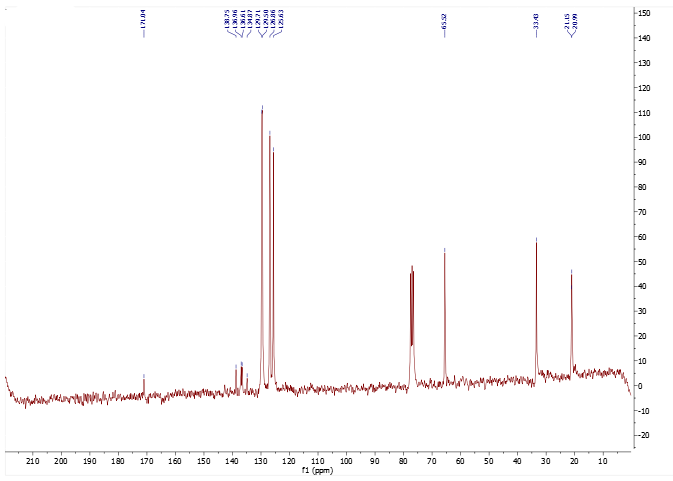


Mass spectrum of 2-(m-Tolyl)-3-(p-tolyl)thiazolidin-4-one (b1):


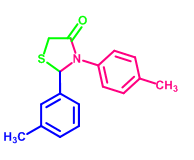

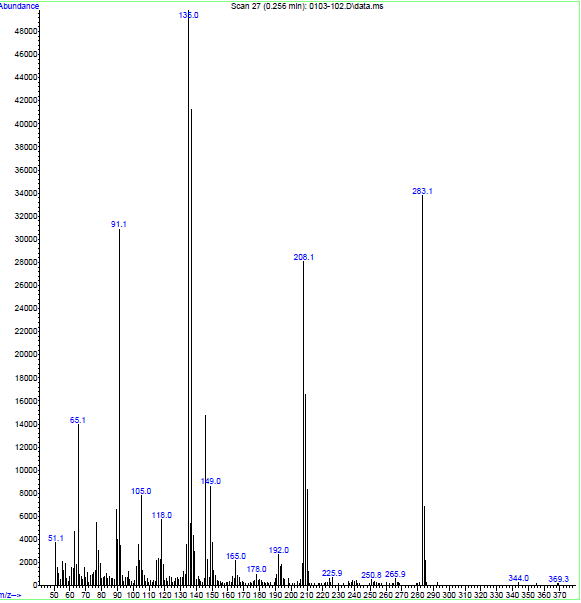


FT-IR spectrum of 2-(3-hydroxyphenyl)-3-(p-tolyl)thiazolidin-4-one (b2):


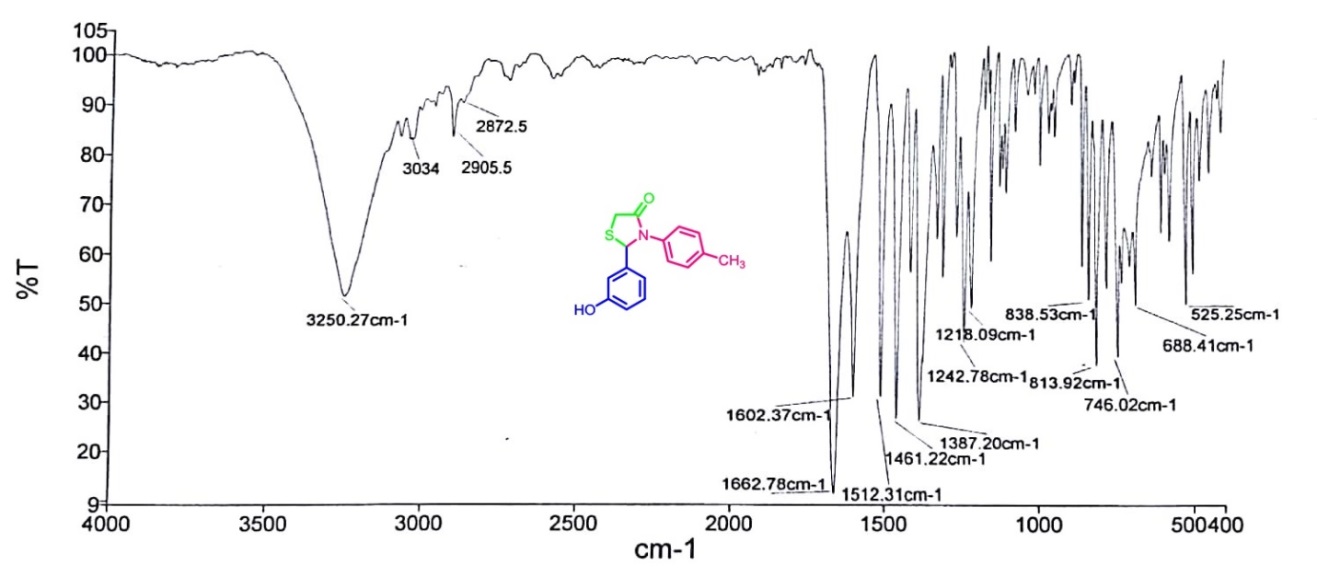


^1^HNMR spectrum of 2-(3-hydroxyphenyl)-3-(p-tolyl)thiazolidin-4-one (b2):


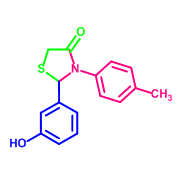


^13^CNMR spectrum of 2-(3-hydroxyphenyl)-3-(p-tolyl)thiazolidin-4-one (b2):


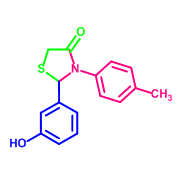

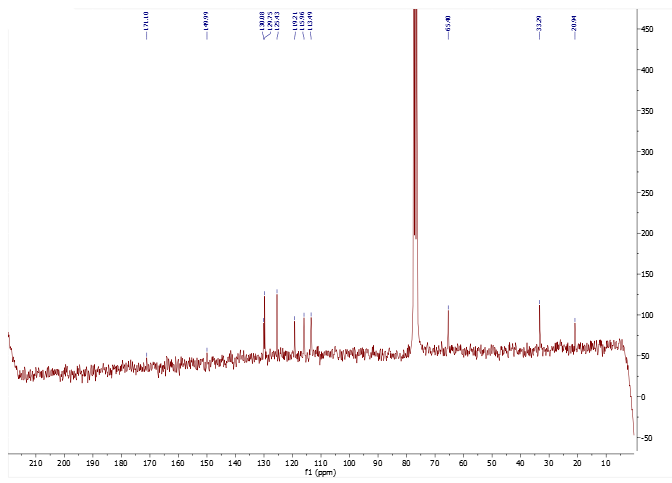


Mass spectrum of 2-(3-hydroxyphenyl)-3-(p-tolyl)thiazolidin-4-one (b2):


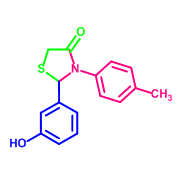

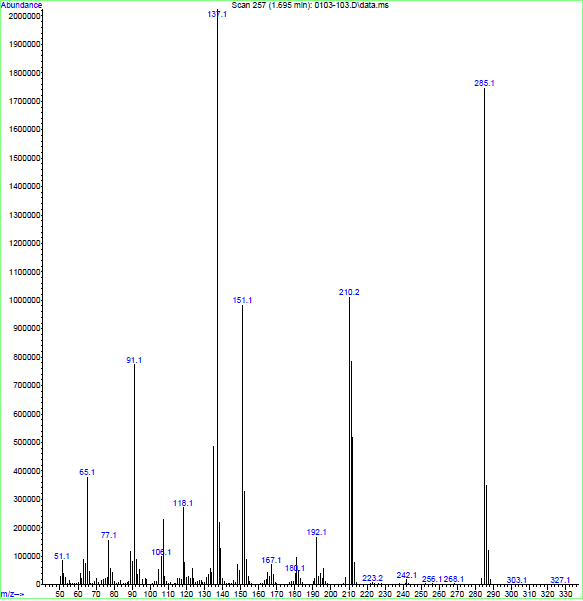


FT-IR spectrum of 2-(4-Chlorophenyl)-3-(p-tolyl)thiazolidin-4-one (b3):


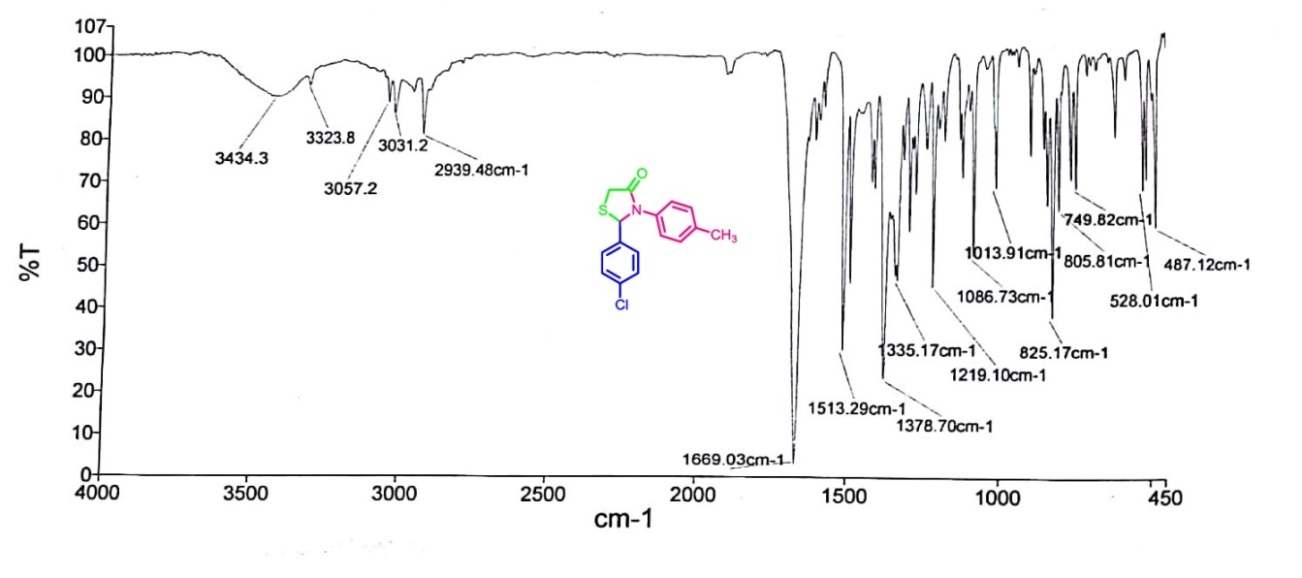


^1^HNMR spectrum of 2-(4-Chlorophenyl)-3-(p-tolyl)thiazolidin-4-one (b3):


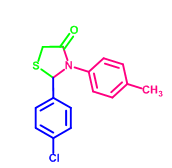


FT-IR spectrum of 2-(3-Nitrophenyl)-3-(p-tolyl)thiazolidin-4-one (b4):


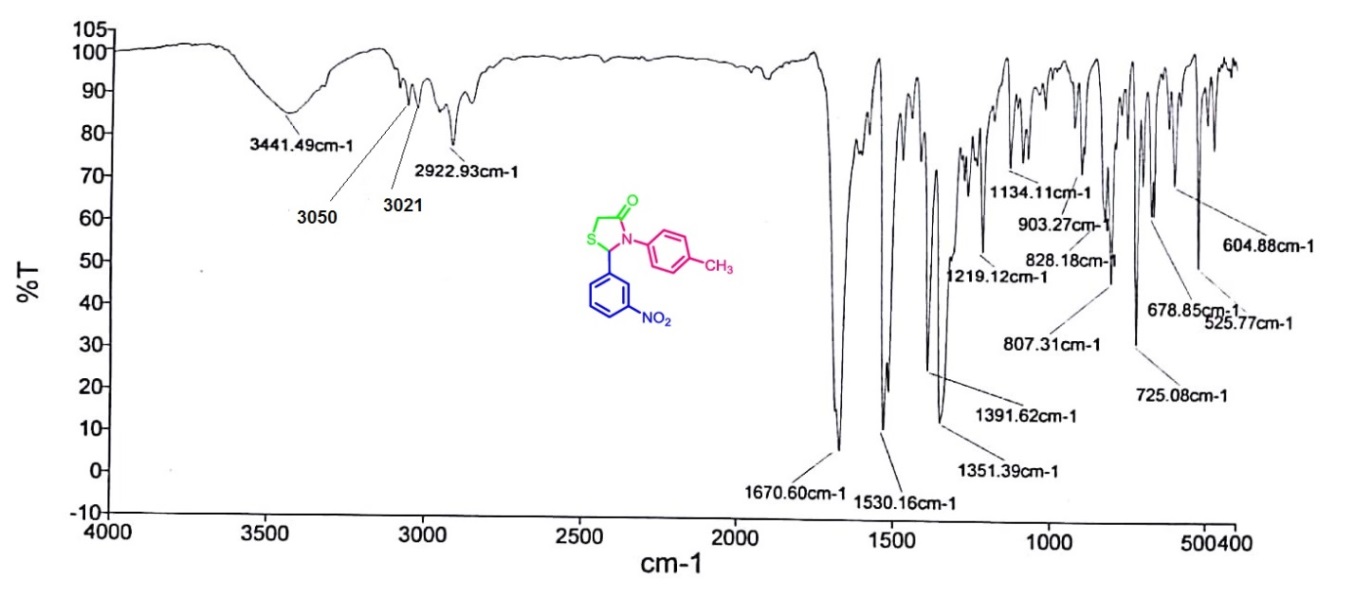


^1^HNMR spectrum of 2-(3-Nitrophenyl)-3-(p-tolyl)thiazolidin-4-one (b4):


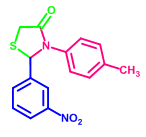


FT-IR spectrum of 2-phenyl-3-(p-tolyl)thiazolidin-4-one (b5):


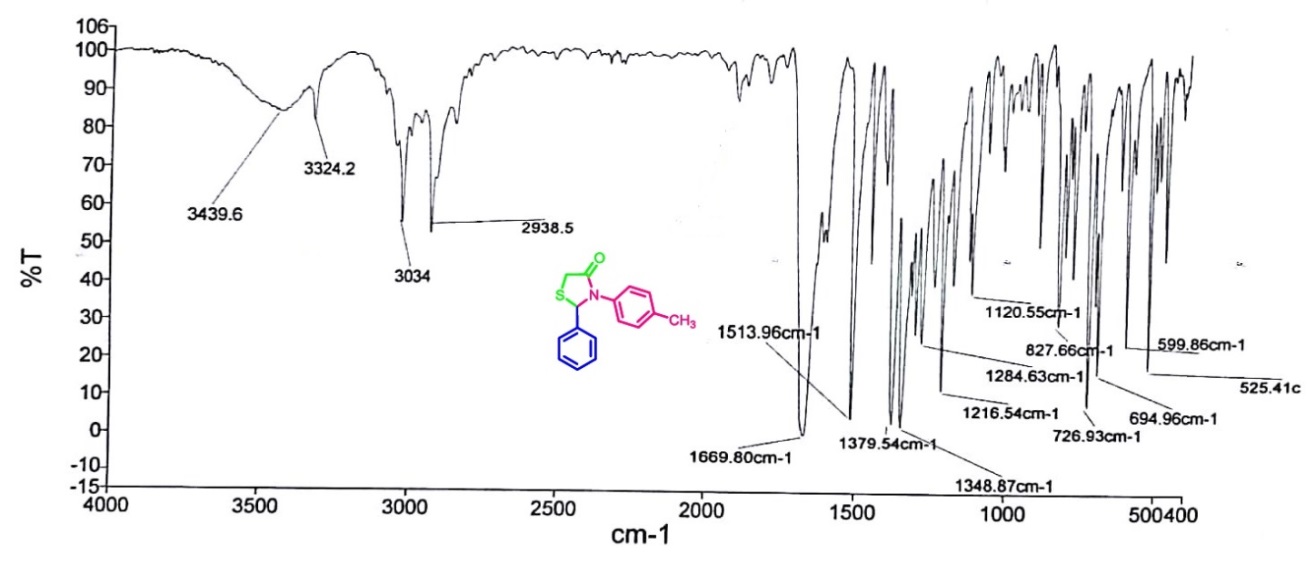


FT-IR spectrum of 2,3-di-p-tolylthiazolidin-4-one (b6):


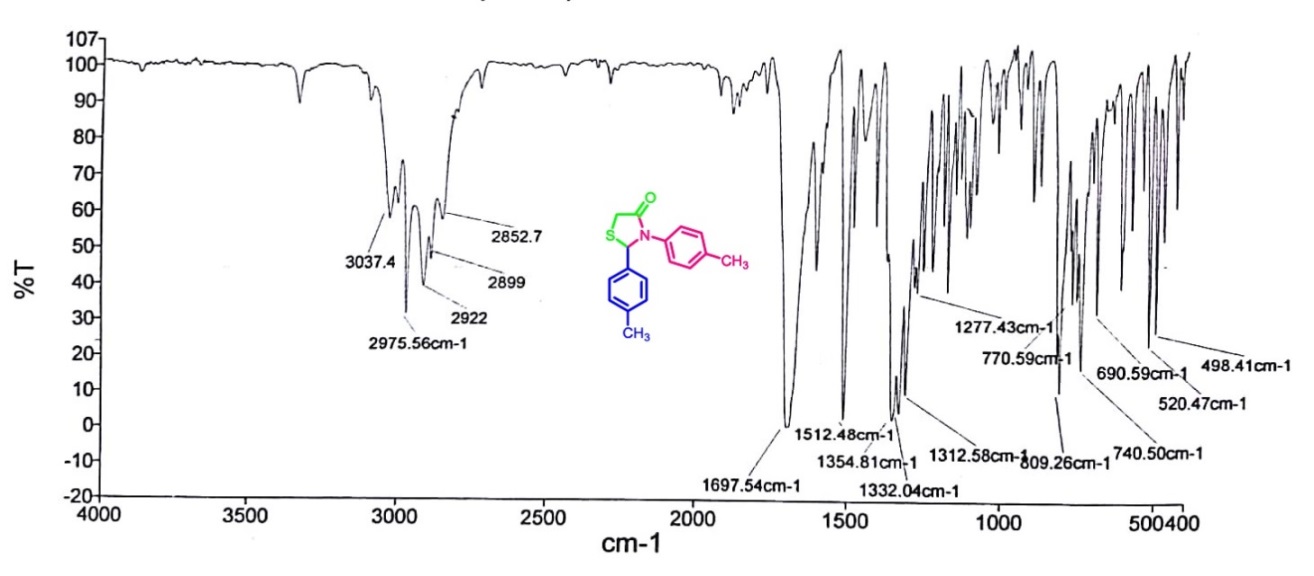


FT-IR spectrum of 2-(2,4-dichlorophenyl)-3-(p-tolyl)thiazolidin-4-one (b7):


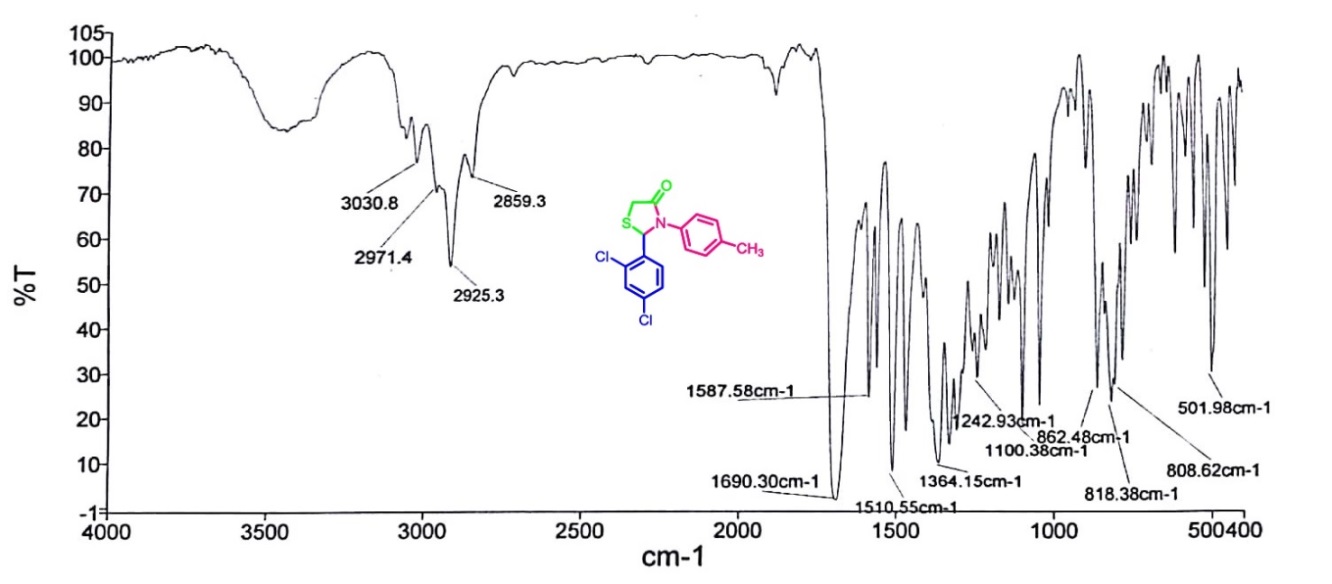


FT-IR spectrum of 2-(pyridin-2-yl)-3-(p-tolyl)thiazolidin-4-one (b8):


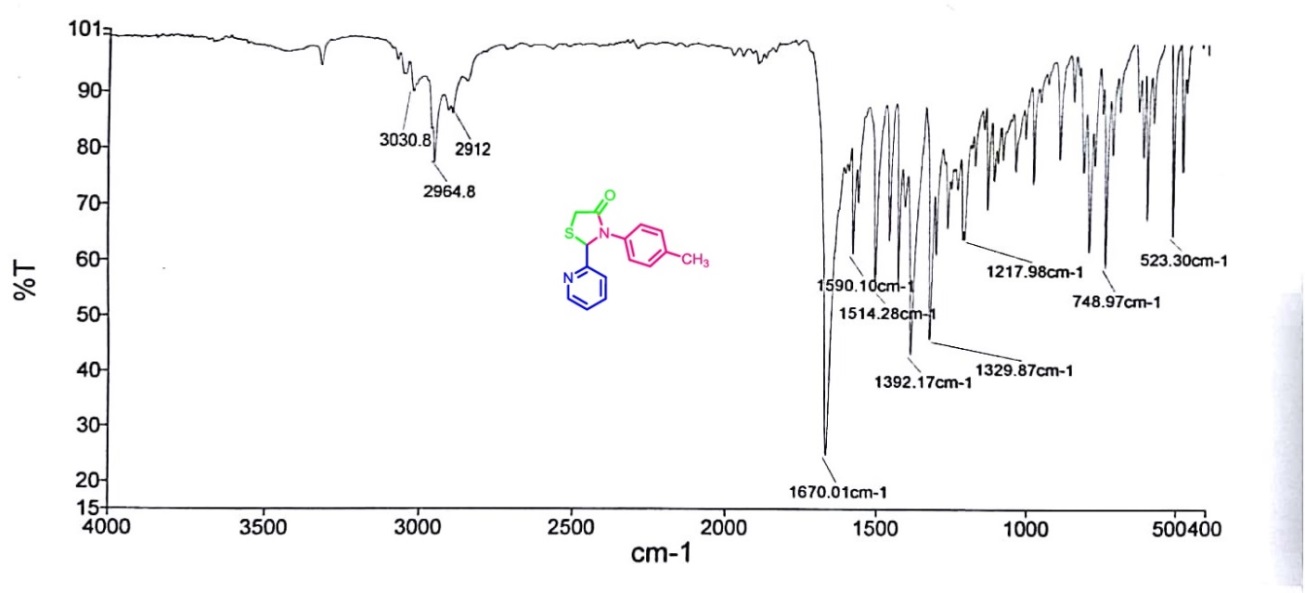


FT-IR spectrum of 2,2'-(1,4-phenylene)bis(3-(p-tolyl)thiazolidin-4-one) (b9):


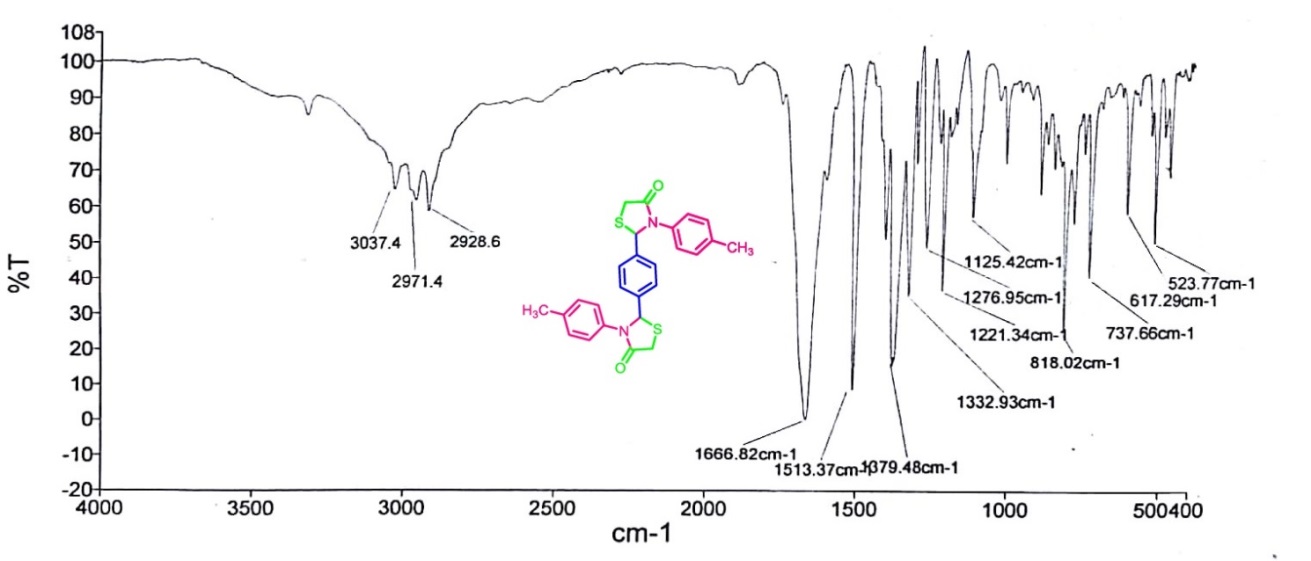


FT-IR spectrum of 2-(4-chlorophenyl)-3-(1H-1,2,4-triazol-5-yl)thiazolidin-4-one (b10):


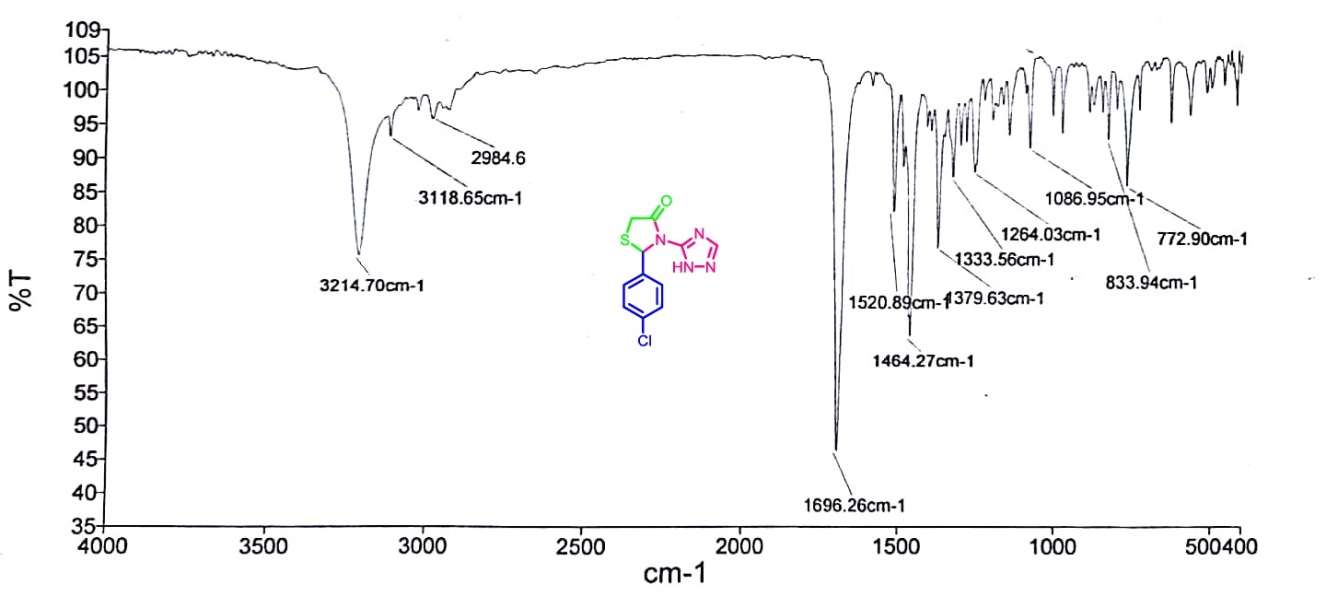


**Experimental**

All reagents were purchased from the Merck and Sigma Aldrich chemical companies and used without further purification. Analytical thin-layer chromatography (TLC) was conducted on pre-coated TLC plates; silica gel 60 F-254 [E. Merck, Darmstadt, Germany]. The ^1^H NMR (250 MHz) and ^13^C NMR (62.5 MHz) were recorded on a Bruker DRX‐400 using CDCl_3_ as a solvent. FT‐IR (KBr) spectra were recorded on an Alpha Perkin Elmer spectrophotometer. Melting points were taken in open capillary tubes with a Stuart melting point apparatus and are uncorrected. Density analysis was done using the AND-HR-200 model solid material density measuring device. The mass spectra were recorded on the Agilent Mass Spectrometer (HP), Model: 5973 Network Mass Selective Detector, Ion source: Electron Impact (EI) 70 eV, Ion source temperature: 230 ºC, with Quadrupole Analyzer.
